# Effects of an integrated poultry value chain, nutrition, gender and WASH intervention (SELEVER) on hygiene and child morbidity and anthropometry in Burkina Faso: A secondary outcome analysis of a cluster randomised trial

**DOI:** 10.1111/mcn.13528

**Published:** 2023-05-27

**Authors:** Aulo Gelli, Anissa Collishaw, Josue Awonon, Elodie Becquey, Ampa Diatta, Loty Diop, Rasmane Ganaba, Derek Headey, Alain Hien, Francis Ngure, Abdoulaye Pedehombga, Marco Santacroce, Laeticia C. Toe, Hans Verhoef, Harold Alderman, Marie T. Ruel

**Affiliations:** ^1^ International Food Policy Research Institute (IFPRI) Washington District of Columbia USA; ^2^ Department of Agricultural and Consumer Economics University of Illinois at Urbana‐Champaign Illinois Urbana USA; ^3^ AFRICSante Bobo‐Dioulasso Burkina Faso; ^4^ Independent Research Consultant Nairobi Kenya; ^5^ Department of Food Technology, Safety and Health, Faculty of Bioscience Engineering Ghent University Ghent Belgium; ^6^ Institut de Recherche en Sciences de la Santé (IRSS) Unité Nutrition et Maladies Métaboliques Bobo‐Dioulasso Burkina Faso; ^7^ Division of Human Nutrition and Health Wageningen University Wageningen Netherlands; ^8^ Division of Human Nutrition Wageningen University Wageningen The Netherlands

**Keywords:** agriculture and nutrition, child malnutrition, impact evaluation, poultry

## Abstract

Nutrition‐sensitive agriculture programmes have the potential to improve child nutrition outcomes, but livestock intensification may pose risks related to water, sanitation and hygiene (WASH) conditions. We assessed the impact of SELEVER, a nutrition‐ and gender‐sensitive poultry intervention, with and without added WASH focus, on hygiene practices, morbidity and anthropometric indices of nutrition in children aged 2−4 years in Burkina Faso. A 3‐year cluster randomised controlled trial was implemented in 120 villages in 60 communes (districts) supported by the SELEVER project. Communes were randomly assigned using restricted randomisation to one of three groups: (1) SELEVER intervention (*n* = 446 households); (2) SELEVER plus WASH intervention (*n* = 432 households); and (3) control without intervention (*n* = 899 households). The study population included women aged 15−49 years with an index child aged 2−4 years. We assessed the effects 1.5‐years (WASH substudy) and 3‐years (endline) post‐intervention on child morbidity and child anthropometry secondary trial outcomes using mixed effects regression models. Participation in intervention activities was low in the SELEVER groups, ranging from 25% at 1.5 years and 10% at endline. At endline, households in the SELEVER groups had higher caregiver knowledge of WASH‐livestock risks (∆ = 0.10, 95% confidence interval [CI] [0.04−0.16]) and were more likely to keep children separated from poultry (∆ = 0.09, 95% CI [0.03−0.15]) than in the control group. No differences were found for other hygiene practices, child morbidity symptoms or anthropometry indicators. Integrating livestock WASH interventions alongside poultry and nutrition interventions can increase knowledge of livestock‐related risks and improve livestock‐hygiene‐related practices, yet may not be sufficient to improve the morbidity and nutritional status of young children.

## Key messages

- Few experimental studies have examined the potential nutritional benefits and health risks of livestock‐related interventions in low‐and middle‐income settings.
- We conducted a 3‐year cluster randomised trial of nutrition‐ and gender‐sensitive poultry intervention in Burkina Faso.
- Despite low participation in the intervention activities, caregiver's knowledge of water, sanitation and hygiene‐livestock risks was higher in the intervention groups than in the control group.
- No effects were found on hygiene and hygiene‐livestock‐related practices (except for practices involving separation between livestock and children) nor on child morbidity symptoms and anthropometry indicators.

## Graphical abstract

We assessed the impact of SELEVER, a nutrition‐ and gender‐sensitive poultry intervention, with and without added water, sanitation and hygiene (WASH) focus, on hygiene practices, morbidity and anthropometric indices of nutrition in children aged 2−4 years in Burkina Faso. A 3‐year cluster randomised controlled trial was implemented in 120 villages in 60 communes (districts) supported by the SELEVER project. Despite low participation in the intervention activities, the caregiver's knowledge of WASH‐livestock risks was higher in the intervention groups than in the control group, but no effects were found on hygiene and hygiene‐livestock‐related practices (except for practices involving separation between livestock and children), nor on child morbidity symptoms and anthropometry indicators.

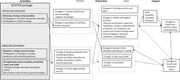

## 1 INTRODUCTION

Despite some progress over the last two decades, child undernutrition remains a public health concern in low‐ and middle‐income countries (Victora et al., 2021). Optimal child nutrition and development result from dietary, caregiving, and health determinants that are shaped by underlying caregiving resources, food security and environmental conditions (Black et al., 2013). The scaling‐up of nutrition‐specific programmes addressing the immediate determinants of malnutrition will be insufficient to meet global targets for improving child nutrition outcomes, and actions across sectors are necessary to accelerate progress (Bhutta et al., 2013; Heidkamp et al., 2021). Nutrition‐sensitive programmes, including integrated agriculture and nutrition programmes, are designed to address the underlying determinants of nutrition, including income, women's empowerment, and the affordability of nutritious foods. These types of multi‐sectoral actions have the potential to accelerate progress in reducing child malnutrition (Ruel & Alderman, 2013).

Integrated agriculture and nutrition programmes have been shown to improve diet diversity and consumption of nutritious foods (Kadiyala et al., 2021; Ruel et al., 2018; Santoso et al., 2021; Sharma et al., 2021). From a nutritional perspective, interventions in livestock are of particular interest due to the high‐quality protein and nutrient density of many animal‐sourced foods (Murphy & Allen, 2003; Neumann et al., 2002). Livestock interventions are increasingly being designed with the goal of improving child nutrition directly through increased consumption of animal‐sourced foods from own production and indirectly through increased income (Ruel et al., 2018). Poultry programmes are relevant due to the contribution of eggs and poultry meat to diets (Iannotti et al., 2014) and the near ubiquity of poultry in low‐income contexts (Guèye, 2000). Despite this potential, there is a dearth of evidence on the role of market‐based poultry−livestock interventions in improving nutrition outcomes (Alderman et al., 2022; Passarelli et al., 2020; Ruel et al., 2018).

Intensification of livestock production may pose child‐health‐related risks through increased exposure to livestock‐related pathogens, resulting in an elevated burden of disease (Penakalapati et al., 2017; Zambrano et al., 2014). These risks are heightened for young children living in low‐income settings characterised by poor water, sanitation and hygiene (WASH) conditions and where livestock is kept in close proximity to household dwellings (Headey & Hirvonen, 2016; Ngure et al., 2019; Schriewer et al., 2015). Poultry is of particular concern, as scavenging systems common in low‐income countries involve poultry roaming in the household compound, exposing young children to ingesting chicken faecal matter (Ngure et al., 2014). Faecal bacteria exposure is associated with environmental enteric dysfunction (EED), a subclinical inflammation of the small intestine responsible for low‐grade chronic immune stimulation associated with child stunting (Humphrey, 2009; Korpe & Petri, 2012; Prendergast et al., 2014). Overnight corralling of livestock in the same room as young children is also associated with elevated markers of EED (George, Oldja, Biswas, Perin, Lee, Ahmed, et al., 2015) and stunting (George, Oldja, Biswas, Perin, Lee, Kosek, et al., 2015; Headey & Hirvonen, 2016).

Little attention has been paid to date in livestock interventions to the prevention of EED through improved poultry–husbandry and hygiene promotion. Similarly, WASH interventions have focused on reducing exposure to human faeces, even though animal faecal matter is arguably more widespread in rural, low‐income contexts (Zambrano et al., 2014). There are several reasons for this evidence gap, including that the biological processes involved in EED are complex and not yet well understood (Mbuya & Humphrey, 2016). These gaps are salient when considering the potential effects of seasonality. Important seasonal variations have been documented on indicators of human diets, health and nutrition and on indicators related to WASH and poultry production systems (Arsenault et al., 2014; Sonaiya & Swan, 2004). This is relevant in the context of rural Burkina Faso, where during the lean, rainy season, households often temporarily leave their compounds and relocate with their young children and livestock to live closer to their fields to tend to the harvest (Gelli, Headey, et al., 2017). These temporary dwellings involve a different WASH environment from that provided in household compounds, modifying the context in which interventions operate. To our knowledge, there is little or no evidence of how the effectiveness of integrated livestock and nutrition interventions varies by season.

In this paper, we address these evidence gaps by assessing the impact of SELEVER (Soutenir l'Exploitation Famaliales pour Lancer l'Elevage des Volailles et Valoriser l'Economie Rurale, or Women's Poultry Programme to Improve Income and Nutrition), a nutrition‐ and gender‐sensitive poultry value chain intervention, with and without additional livestock WASH behaviour change, to improve household hygiene practices, reduce child morbidity and improve anthropometric indices of nutrition status in young children in rural Burkina Faso. We also examine whether treatment effects varied by season. This analysis was pre‐specified in the SELEVER trial protocol and focuses on child morbidity and child anthropometry, both secondary outcomes of the trial; the analysis also includes a seasonal WASH substudy (Gelli, Becquey, et al., 2017). The results of the other trial outcomes are reported separately (Becquey et al., 2022; Leight, Awonon, Pedehombga, Ganaba, Gelli, 2022; Leight, Awonon, Pedehombga, Ganaba, Martinez, et al., 2022). Briefly, the intervention was found to have little effect on households' poultry production or profits, though there is some evidence that larger producers increased their input use and reduced poultry mortality. In terms of diet quality, children in SELEVER intervention groups were somewhat more likely to consume eggs, while women were more likely to have increased the probability of adequacy of iron intake. No effects were observed for other measures of diet quality.

## 2 METHODOLOGY

### 2.1 Study design and participants

The study protocol and design of the SELEVER trial have been previously reported and are available online (Gelli, Becquey, et al., 2017). Briefly, the SELEVER trial was a cluster‐randomised controlled trial implemented in 120 rural villages within 60 communes (districts) supported by SELEVER in the Boucle de Mouhoun, Centre‐Ouest and Haut−Bassins regions of Burkina Faso. The intervention was implemented by Tanager (implementing nongovernmental organisation, NGO), and it targeted rural and peri‐urban communities within the three targeted regions on the basis of a set of variables related to poultry production and market access. The most recent nutritional surveillance survey in Burkina Faso found rates of stunting and wasting in children <5 years 21% and 9%, respectively, and particularly poor levels of infant and young child feeding (IYCF) practices (DNMS 2016). In the latest Burkina Faso Demographic and Health Survey (DHS), 80% of households across the country owned poultry, yet only 14% of children 6−24 months had consumed poultry flesh, and just 3% had consumed eggs in the past 24 h (INSD/Burkina Faso & ICF International, 2012).

The two primary reference age groups in the SELEVER trial included women aged 15−49 years with at least one child aged 2−4 years at baseline and their (index) child aged 2−4 years at baseline. Women were eligible for inclusion if they resided permanently in the targeted villages, were aged 15−49 years and had at least one (index) child aged 2−4 years living in the same household. A secondary reference age group, including index children's younger siblings (aged 6−24 m at baseline), was used for exploratory analyses. The Comite de Recherche en Sante MS/MRSI in Burkina Faso (N°2016‐12‐142) and the Institutional Review Board of the International Food Policy Research Institute DC (approved 26/12/2016, ref: IRB00007490) approved the study protocol. All participants provided written informed consent prior to the survey interviews.

### 2.2 Randomisation and masking

**Figure 1:**
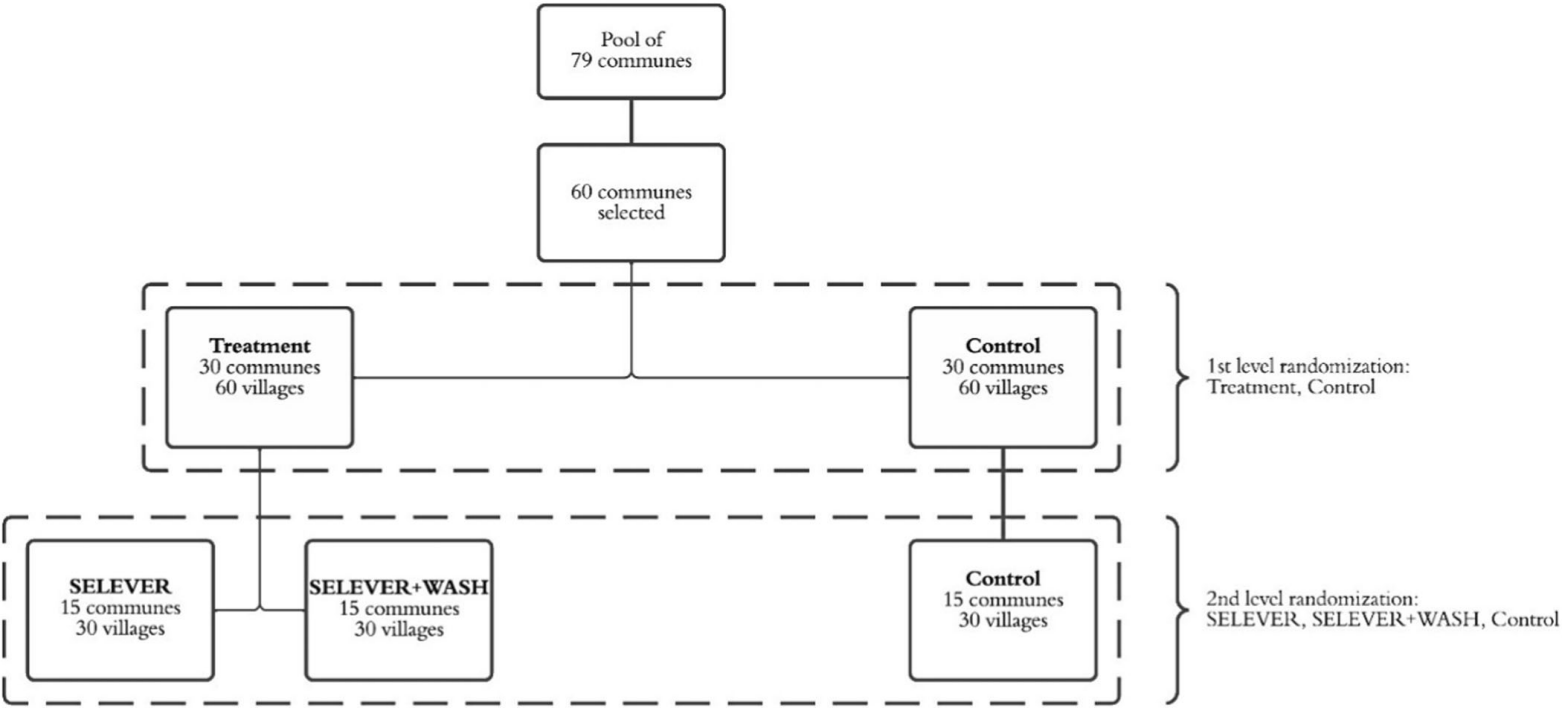
Schematic view of the randomisation for the SELEVER trial and WASH substudy. WASH, water, sanitation and hygiene.

Communities were randomly assigned in a two‐stage procedure to one of three treatment arms: SELEVER intervention group, SELEVER + WASH intervention group and a control group with no intervention for at least the 3 years of the study duration (Figure 1). For logistical reasons, the SELEVER intervention was implemented at the commune level. During the preparation stages of the trial, 60 communes were selected from a pool of 79 communes available for scale‐up in the targeted regions. The criteria for commune selection were: (1) not included in the SELEVER pilot communes; (2) classified in the national census as rural or periurban; (3) all‐year accessibility by road; and (4) for the communes in the Hauts–Bassins region, proximity to the Boucle de Mouhoun and Centre Ouest regions. To achieve balance, the first stage randomisation allocated the 60 communes in the study into two groups (SELEVER and control) using a restricted randomisation procedure that modelled selection using commune‐ and village‐level variables obtained through the national census of 2006, including population size, the existence of a government centre, number of female associations, main agricultural crop, main source of revenue, market access/presence, health centre presence, number of functional boreholes and number of functional wells. An algorithm was developed using Stata to randomly allocate communes to two different groups stratified by region and then select two villages in each commune from the list of available villages. Villages that were too small to allow for a survey sample to be drawn (less than 15 households with children in the 2–4 year age group based on the latest DHS demographics) or too large to be considered rural (with a population over 5000 people, or over the 95 percentile of the population distribution) were excluded from the list. The algorithm then regressed the selection into the treatment group based on the village‐ and commune‐level variables over 3000 allocations, selecting the permutation that minimised the *r* ^2^ statistic for the predicted selection. The second stage randomisation assigned the 30 SELEVER village pairs (one pair for each commune) to either the SELEVER or SELEVER + WASH and a subsample of 15 communes from the control group for the seasonal substudy, using a similar restricted randomisation procedure. Masking of participants was not possible because of the obvious differences between interventions.

### 2.3 Procedures

The SELEVER project was designed by Tanager and implemented in partnership with in‐country NGOs, private institutions and government services in Burkina Faso. SELEVER aimed to improve poultry production and the diets and nutritional status of women and children in the targeted regions. Project implementation involved three main components focussing on poultry revenue generation, diets and nutrition and women's empowerment domains. Intervention activities included training and behaviour change communication (BCC), with no financial or in‐kind transfers for participants. The nutrition component included BCC on improved dietary and nutrition practices provided through women's groups by local NGOs trained by Tanager. The content of the BCC included the promotion of improved diets and basic hygiene practices. This included messaging on IYCF practices, diet diversification and hygiene‐related practices (including a total of 30 topics divided into 3 modules). The BCC materials promoted daily consumption of at least three key food groups, including energy‐giving foods (starchy staples and fats), protective foods (fruits and vegetables) and protein‐rich, body‐building foods (including animal source foods, legumes and nuts). The gender component, implemented alongside the nutrition activities, included sensitisation on women's empowerment through monthly community‐level training sessions, follow‐up home visits, peer‐group support and advocacy conducted by high‐profile community members such as religious or traditional community leaders and women leaders. The training curriculum focussed on strengthening women's role in decision‐making on topics including entrepreneurship, nutritious food production, consumption and sales, as well as on child health, feeding and care practices.

The poultry component involved a set of training practices on poultry husbandry, including improved housing, vaccinations, feed, financing and marketing practices provided by micro‐finance institutions trained by Tanager. This curriculum was delivered to micro‐finance group members through 8 videos, typically screened over a series of 3 meetings. In addition, SELEVER trained village volunteer extension agents (VVVs) to improve the quality of their services, with a focus on poultry vaccination, to reduce mortality.

The additional WASH behaviour change intervention aimed to enhance the impact of the SELEVER programme on children's health and nutrition status by improving the general WASH environment at the community and household level and specifically reducing the risk of exposure to livestock faeces for young children. In the SELEVER‐WASH arm, a poultry–livestock lens was applied to the Community‐Led Total Sanitation (CLTS) behaviour change approach (Chambers & Kar, 2008). The CLTS activities were based on materials developed in the context of the national strategy currently being rolled out across Burkina Faso. The CLTS approach generally involves three stages, including (1) pretriggering (engagement with community members); (2) triggering (conducting group meeting activities to elicit emotional responses, including shame and disgust, and generate motivation to eliminate open defecation); and (3) follow‐up (monitoring progress and feedback towards eliminating open defecation) in the community. The roll‐out of the community‐level activities undertaken in these 3 stages was broadened to also include livestock‐ and poultry‐specific topics. In pretriggering, this included additional engagement with poultry and livestock‐related actors. The presence of poultry and livestock faeces was included as explicit themes in the triggering activities, leading to the development of an action plan. Following the triggering event, facilitators were trained, and a village hygiene committee was organised, including youth, women and other resource people with influence in the community. Committee members conducted home visits to follow up on the planned activities, providing feedback and advice to NGO staff.

Prior to the baseline survey, a household census was conducted in the targeted villages, including information on basic demographics and poultry flock size. Data from the census were used to construct a listing of households with women aged 15−49 years with children in the 2−4 years age group for the survey sample, stratified by larger/smaller poultry producers (with larger producers defined as those owning a poultry flock of over 20 birds). A sample of 15 poultry‐producing households was drawn to form the main study population (6 from larger poultry‐producing households). A second sample of 12 out of the 15 households was drawn to select participants for the pre‐specified seasonal substudy (with 5 out of 12 households drawn from larger producer strata). During the baseline survey interview, an index child in the 2−4 years age range was randomly selected for inclusion in the biomedical component of the analysis alongside their primary female caregiver.

Data collection was performed using Computer Assisted Personal Interview surveys designed using SurveyBe software running on Android and Microsoft Windows tablets. Survey forms were written in French, and all enumerators also spoke the local languages. The survey included data on sociodemographic characteristics; IYCF, nutrition, health and hygiene practices and knowledge of caregivers; as well as a wide range of indicators at village, household, caregiver and child level. Intervention exposure was measured by the self‐reported recall of participation in activities related to the SELEVER intervention, including participation in group activities and follow‐up home visits. Information on programme participation over the previous 12 months was collected separately for index women, their husbands and any other household members. Individual responses were aggregated to obtain household‐level estimates of exposure (using means for continuous variables or Boolean OR operation for binary variables). Child weight was measured to the nearest 100 g using an electronic scale (SECA 876, Germany). The recumbent length of children <2 years of age and the standing height of children >2 year of age was measured to the nearest 0.1 cm using portable fixed base stadiometers or length boards (SECA 417). All measurements were taken in duplicate during the same session by an anthropometrist and an assistant and were practiced before the survey through standardisation exercises. From the standardisation sessions, inter and intraobserver variations of measurement error were documented, and the necessary corrections to procedures were made prior to data collection. Index child morbidity was assessed through caregiver recall of symptoms during the previous 7 days. Symptoms included vomiting, fever, cough, respiratory difficulties, blood in stool, liquid or semi‐liquid stools, other types of diarrhoea, or other illnesses.

**Figure 2:**
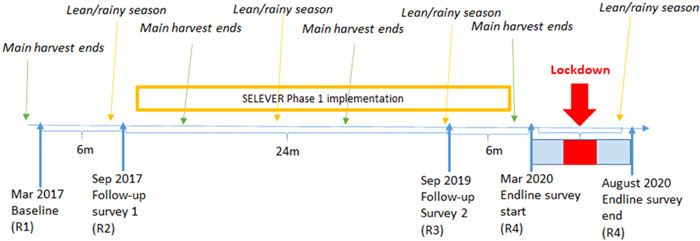
SELEVER study timeline in Burkina Faso.

The trial included four rounds of data collection (including 2 preintervention rounds timed in different seasons) between 2017 and 2020, with a mixed‐methods process evaluation at midterm (Figure 2). The baseline survey was conducted between March and June 2017, the lean‐season follow‐up survey 1 was conducted between September−November 2017, and lean‐season follow‐up survey 2 was conducted between September−November 2019. The endline survey began on the 7th of March 2020 as planned, just before the COVID‐19 pandemic (WHO, 2023). However, data collection was paused by the principal investigator on the 24th of March after consultation with the in‐country research partner and the SELEVER trial steering group chair, days before the national lockdown was mandated. Authorisation was received to restart the survey on the 9th of June from the IFPRI ethics review board, IFPRI senior management, the local IRB, and in‐country health officials. Data collection resumed after a refresher enumerator training and was completed in the first week of August (Figure 2). Further details of measures taken in response to the COVID‐19 pandemic are outlined in the Annex.

### 2.4 Outcome measures

**Figure 3:**
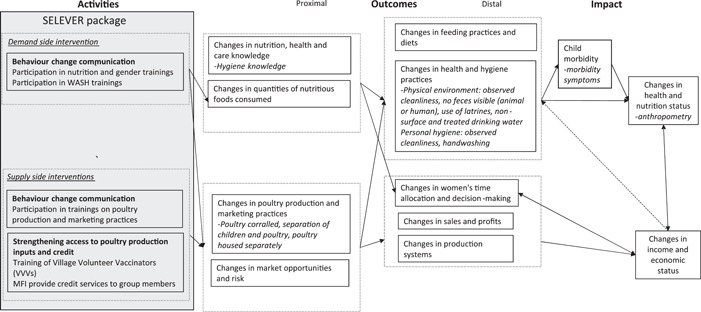
Programme impact pathways for the SELEVER intervention and child anthropometry outcomes.

The child morbidity and anthropometric indicators reported here are prespecified as secondary outcomes in the SELEVER trial protocol. An aggregate index of morbidity symptoms was calculated using the sum of all symptoms reported. Anthropometric indicators included children's height, weight, height‐for‐age z‐score and body mass index z‐score (BMIZ), the prevalence of stunting (HAZ < −2) and of thinness (BMIZ < −2). HAZ scores were calculated using the 2006 WHO growth standards for children <5 years and the 2007 WHO growth reference for children >5 years (de Onis, 2007; WHO, 2006). The main trial population was assessed at baseline and endline, whilst the seasonal substudy population was assessed in all 4 survey rounds. A range of intermediate outcomes on the programme impact pathways (PIPs) was also measured, including hygiene knowledge and indicators on the WASH and livestock−WASH environment (Figure 3). Maternal and paternal hygiene knowledge scores (range 0−18) were constructed using responses to questions about the risks associated with cohabitation with livestock and poultry, ways to avoid risks posed by close proximity to livestock and poultry, the risks associated with open defecation, ways to overcome open defecation risks and handwashing recommendations. The WASH indicators include scores generated by aggregating responses to questions about the WASH environment and behaviours that were specifically targeted by the SELEVER intervention. The WATER score (range 0 to 3) indicates whether the primary source of drinking water was surface water, whether households did not treat drinking water via chlorination or filtration and whether animals had no access to the primary source of drinking water. The HYGIENE score (range 0−10) indicates whether households did not have a handwashing facility or soap and observations about the cleanliness of the household compound, including the general appearance of the compound, cleanliness of latrines, absence of garbage and soiled children's underwear, absence of visible human faeces, absence of visible chicken faeces in the yard or kitchen area and whether animals were free‐roaming. The SANITATION score (ranging from 0 to 3) indicates whether households had a functioning latrine, whether the latrine was built in concrete and whether there was a slab present. The overall WASH score was generated by summation of the WASH scores. All these aggregate indicators were coded such that higher values indicate a positive or preferred outcome. Three variables were used to assess the degree of separation between children and three categories of livestock (poultry, small livestock and large livestock)^1^. For each livestock category, the indicator is a sum of responses to questions about whether the child had access to where the animal spent most of its time, whether the animal was kept inside the house, and whether the distance between the animal and the child at night was less than 10 m. As in the case of the WASH variables, higher values indicate better outcomes (i.e., low values represent close contact between animals and children, while higher values represent a greater degree of separation between animals and children). Supporting Information: Table A1 provides a description of the constructed scores.

### 2.5 Statistical analysis

The sample size calculations for the related primary outcomes of the trial were for 1080 index children, based on a minimum detectable effect size of 0.08 for the trial primary outcome (mean probability of adequacy of nutrient intake) at 80% statistical power, *α* = 0.05 and intracluster correlation coefficient (ICC) = 0.05. Calculations were performed using data from an observational study examining food intake in two of the three targeted regions (Centre‐Ouest and Boucle du Mouhoun) (Arsenault et al., 2014; Martin−Prevel et al., 2016). The analysis was intended to treat using single difference analysis of covariance (ANCOVA) regression models with standard errors clustered at cluster (commune) level, accounting for the sampling weights, and a binary variable representing the main effect of the SELEVER intervention (SELEVER groups compared to the control group) in the main trial comparisons, and a second binary variable for the second level substudy comparisons for the SELEVER + WASH intervention (comparing SELEVER and SELEVER + WASH groups to the control group). In adjusted analyses, we also controlled for baseline covariates, including child age and gender, and pre‐intervention values of household variables not balanced at baseline. As the COVID lockdown‐induced delays introduced a mismatch between baseline (March−June) and endline (March−August) survey periods, additional controls were also added for the survey month postlockdown. Unless otherwise specified, the regressions used linear regression models for continuous variables and Poisson models for count variables. For interpretation, impacts were considered statistically significant at *p* < 0.05 and marginally significant at *p* < 0.1. All analyses were performed using Stata. The trial was registered on the ISRCTN registry on 2 December 2016.

### 2.6 Role of the funding source

The funders of the study had no role in the data collection, analysis, interpretation or writing of the manuscript. The corresponding author had full access to all study data and had the final responsibility to submit the manuscript for publication.

## 3 RESULTS

### 3.1 Baseline characteristics and loss to follow‐up

**Figure 4:**
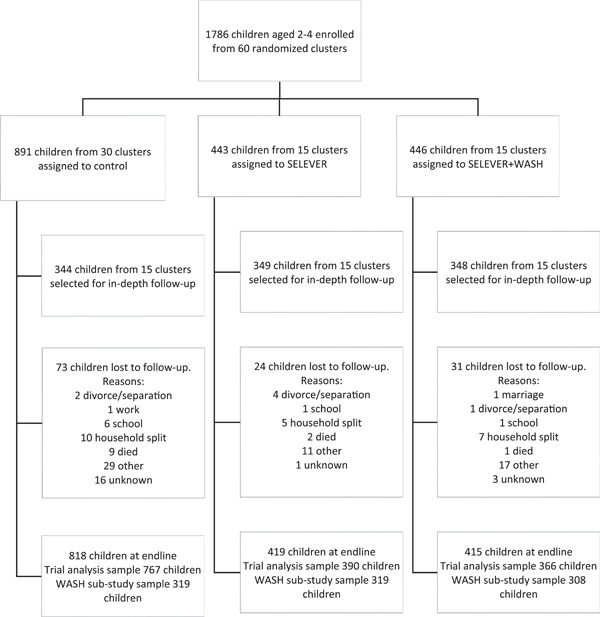
Trial profile notes: 120 clusters were randomly assigned. After randomisation, one village allocated to the SELEVER + WASH intervention was excluded as the intervention was not implemented there due to a mismatch with programme implementers. WASH, water, sanitation and hygiene.

The baseline survey included a total of 1,786 households in 199 villages from 60 communes across the three targeted regions of Burkina Faso (Figure 4). At baseline, less than 10% of household heads had completed primary education, over 40% of households were polygamous and over three quarters lived below the 1.90 USD per capita (PPP) poverty line. The general WASH environment was poor. Over 30% of households used unimproved water sources, and over half of households had no latrines within the compound. Human faeces were visible in over a tenth of household compounds, while animals were free roaming and chicken faeces visible in over two‐thirds of household compounds. The proportion of household heads who completed primary education was higher, and of polygamous households lower in the control group compared to the SELEVER group (Table 1). In the WASH substudy population, households in the SELEVER + WASH group appeared to have substantively lower WASH levels than those in control and SELEVER groups across all WASH sub‐domains examined. Other household and maternal characteristics were similar across treatment groups. The endline survey in 2020 included 1671 households, leading to a 7% household attrition rate over the 3‐year study period. This level of loss to follow‐up is in line with the expectations from a longitudinal survey design of this duration. Loss to follow‐up was not significantly different across SELEVER and control groups. No statistically significant differences at baseline were found in the outcomes of children lost to follow‐up.

**Table 1:** Household, maternal and child level characteristics at baseline, SELEVER trial and WASH‐substudy populations.

	Trial		WASH substudy		
	Control	SELEVER	Control	SELEVER	SELEVER + WASH
Household characteristics					
Age head of household, years	41.47 (24.05)	43.74 (21.47)	41.94 (25.79)	42.95 (20.92)	44.55 (21.88)
Household head completed primary education	0.11	0.06	0.11	0.07	0.04
Household size, *n*	8.08 (8.22)	8.61 (7.56)	8.31 (8.88)	8.28 (8.07)	8.94 (7.06)
Polygamous household	0.40	0.46	0.40	0.44	0.48
Food security (HFIAS) Score (0−27)	2.96 (9.49)	2.98 (8.24)	3.67 (8.58)	2.85 (8.29)	3.10 (8.63)
Poverty headcount, PPP $1.90	0.80	0.78	0.79	0.76	0.80
WASH characteristics					
WASH environment score (0−16)	13.22 (6.30)	13.33 (7.69)	13.14 (7.86)	13.78 (9.15)	12.87 (5.45)
Water score (0−3)	1.17 (1.96)	1.18 (1.97)	1.26 (1.54)	1.30 (1.64)	1.05 (2.06)
Non‐surface drinking water	0.64	0.69	0.73	0.74	0.64
Household treats water	0.12	0.09	0.12	0.11	0.07
Animals cannot access drinking water	0.41	0.39	0.41	0.44	0.34
Sanitation score (0−3)	0.95 (2.99)	0.87 (2.72)	0.92 (3.32)	1.09 (3.20)	0.64 (1.68)
Own a working latrine	0.45	0.40	0.42	0.47	0.33
Latrine built in concrete	0.20	0.19	0.20	0.25	0.13
Slab present and in good condition	0.30	0.28	0.31	0.37	0.19
Hygiene score (0−10)	4.47 (4.41)	4.43 (4.33)	4.47 (5.24)	4.52 (5.48)	4.33 (2.92)
Handwashing facility	0.04	0.03	0.03	0.04	0.02
Have soap	0.02	0.00	0.01	0.01	0.00
Concession appears clean	0.41	0.42	0.39	0.48	0.37
No open garbage in concession	0.39	0.42	0.37	0.42	0.42
Animals contained	0.72	0.71	0.78	0.70	0.73
Clean toilets	0.26	0.28	0.25	0.33	0.22
No human faeces visible	0.88	0.87	0.86	0.86	0.88
No soiled diapers	0.79	0.79	0.79	0.77	0.80
No chicken faeces in concession	0.33	0.26	0.33	0.27	0.24
No chicken faeces in kitchen	0.63	0.65	0.65	0.64	0.65
Separation of animals & children score (0−9)	6.63 (2.60)	6.85 (2.39)	6.49 (2.49)	6.87 (2.34)	6.84 (2.52)
Child‐small livestock (0–3)	2.13 (1.27)	2.27 (0.97)	2.09 (1.21)	2.27 (0.94)	2.27 (1.03)
Child‐livestock (0–3)	2.51 (0.86)	2.50 (0.88)	2.44 (0.81)	2.49 (0.83)	2.50 (0.96)
Child‐poultry (0−3)	1.98 (0.95)	2.09 (1.20)	1.95 (1.00)	2.11 (1.07)	2.08 (1.36)
Mother's cleanliness score (0−4)	2.87 (1.71)	2.70 (2.16)	2.93 (1.72)	2.72 (2.38)	2.68 (1.98)
Appearance of mother's hands are clean	0.75	0.69	0.75	0.70	0.68
Appearance of mother's hair is clean	0.62	0.60	0.64	0.61	0.59
Appearance of mother's clothing is clean	0.71	0.66	0.71	0.66	0.65
Appearance of mother's face is clean	0.80	0.75	0.82	0.74	0.76
Child's cleanliness score (0‐4)	1.62 (2.23)	1.38 (2.33)	1.70 (2.29)	1.52 (2.50)	1.22 (1.90)
Appearance of child's hands are clean	0.32	0.24	0.31	0.29	0.18
Appearance of child's hair is clean	0.47	0.43	0.50	0.44	0.42
Appearance of child's clothing is clean	0.35	0.29	0.39	0.33	0.24
Appearance of child's face is clean	0.48	0.42	0.50	0.46	0.37
Mother characteristics					
Height (cm)	161.39 (8.48)	161.56 (11.53)	161.18 (8.03)	160.99 (14.32)	162.15 (5.79)
Weight (kg)	57.61 (16.97)	56.71 (10.18)	56.78 (13.00)	56.75 (11.21)	56.66 (9.38)
BMI	21.88 (4.30)	21.54 (3.37)	21.79 (4.07)	21.53 (3.78)	21.55 (3.04)
Index child characteristics					
Child is male	0.53	0.51	0.53	0.48	0.53
Age, months	40.48 (13.69)	41.15 (10.19)	39.87 (11.40)	41.19 (10.32)	41.10 (10.46)
Morbidity score (0‐6)	0.16 (0.70)	0.14 (0.54)	0.15 (0.51)	0.18 (0.56)	0.10 (0.41)
Vomiting	0.00	0.01	0.00	0.01	0.00
Fever	0.08	0.05	0.07	0.05	0.04
Cough	0.03	0.04	0.04	0.05	0.02
Respiratory difficulties	0.01	0.01	0.01	0.01	0.00
Diarrhoea	0.02	0.04	0.02	0.04	0.03
Other illness	0.01	0.01	0.01	0.01	0.00
Height, cm	92.87 (10.74)	93.64 (7.64)	92.44 (11.25)	93.28 (6.79)	94.04 (7.57)
Weight, kg	13.07 (3.01)	13.12 (2.12)	12.94 (2.90)	13.11 (2.24)	13.12 (2.06)
Height for age z‐score (HAZ)	−1.34 (1.33)	−1.22 (1.33)	−1.37 (1.68)	−1.32 (1.16)	−1.12 (1.19)
BMI for age z‐score (BMIZ)	−0.32 (1.36)	−0.48 (1.38)	−0.34 (1.61)	−0.38 (1.44)	−0.58 (1.11)
Stunting (HAZ < −2)	0.25	0.22	0.25	0.25	0.19
Thinness (BMIZ < −2)	0.03	0.05	0.03	0.06	0.05
Households, *n*	899	878	453	446	432

*Note*: This table presents baseline values of key household, WASH, maternal and index child characteristics in the different treatment arms. The data are presented as means with standard deviations in parentheses for continuous variables or as proportions for binary variables. The SELEVER trial corresponds to the first level of randomisation, while the WASH substudy corresponds to the second level of randomisation.

Abbreviations: BMI, body mass index; HAZ, height for age z‐score; HFIAS, household food insecurity access scale; PPP, purchasing power parity; WASH, water, sanitation and hygiene.

### 3.2 Programme uptake

We report programme uptake at two time points, including endline (3+ years post baseline) for the main trial population and during the lean season follow‐up (2.5 years post‐baseline) for the WASH substudy subpopulation. Chronologically, the lean season follow‐up was carried out just prior to the completion of the SELEVER implementation cycle whilst the endline was conducted after the 3‐year implementation had ended. At endline, only 10% of households in the SELEVER group (vs. 1% in the control group) had attended training on poultry, nutrition, gender and WASH in the 12 months prior to the survey, with an average number of 1.8 in the training attended (vs. 0.8 in the control group) (Table 2). In the SELEVER group, one‐time participation was highest for the WASH‐related training (31%), followed by participation in the training related to poultry (26%), nutrition (20%) and gender (12%). Participation intensity in the SELEVER group was low but fairly even across components, with approximately 0.5 training sessions on average for each of the 3 programme components over the previous 12 months. Follow‐up household visits were most common for WASH‐related counselling (13%), followed by nutrition (6%) and gender (3%). Exposure during the lean‐season follow‐up was as expected as it coincided with the peak programme implementation period, higher than that observed at endline (Table 3). Self‐reported attendance in all SELEVER programme components in the previous 12 months was 25% in the SELEVER group (2% in the control group), with respondents reporting having attended 4.3 training sessions on average (compared to 1.2 in the control group). Exposure by component was highest for WASH activities (49%, average of 1.2 training sessions), followed by poultry activities (44%, average of 1.5 training sessions) and nutrition and gender activities (34%, 1.6 training sessions attended). During both the endline and lean season rounds, there were no significant differences in exposure between SELEVER and SELEVER + WASH groups, though the results of the pooled analysis across the two rounds suggest that WASH exposure was higher in the SELEVER + WASH group compared to the SELEVER group (not reported). These results also suggest that some element of cross‐over between SELEVER and SELEVER + WASH had occurred on the WASH‐livestock messaging, as first highlighted during the process evaluation (Gelli et al., 2019).

**Table 2:** SELEVER programme uptake at endline, SELEVER trial and WASH‐substudy populations.

	Trial		*p* Value	WASH substudy			*p* Value Δ		
Variable	(1) Control	(2) SELEVER	(1)−(2)	(1) Control	(2) SELEVER	(3) SELEVER + WASH	(1)−(2)	(1)−(3)	(2)−(3)
Attended training on poultry, nutrition, gender and WASH	0.01	0.10	<0.001	0.02	0.10	0.09	<0.001	<0.001	0.583
Total number of training sessions attended, *n*	0.79 (0.11)	1.75 (0.12)	<0.001	0.67 (0.13)	1.68 (0.16)	1.82 (0.18)	<0.001	<0.001	0.537
Proportion who attended any poultry training (s)	0.06	0.26	<0.001	0.06	0.25	0.27	<0.001	<0.001	0.37
Number of poultry training sessions attended, *n*	0.10 (0.02)	0.59 (0.05)	<0.001	0.09 (0.02)	0.59 (0.06)	0.58 (0.08)	<0.001	<0.001	0.923
Proportion who received VVV services	0.6	0.66	0.132	0.65	0.63	0.70	0.024	−0.047	0.152
Proportion who attended any nutrition training (s)	0.11	0.2	<0.001	0.11	0.18	0.21	−0.066	−0.1	0.407
Proportion who attended any gender training (s)	0.04	0.12	<0.001	0.03	0.13	0.10	<0.001	<0.001	0.187
Number of nutrition/gender trainings attended, *n*	0.3 (0.05)	0.57 (0.06)	0.001	0.3 (0.08)	0.57 (0.07)	0.57 ([0.10])	−0.268	−0.268	0.994
Proportion receiving any nutrition home visit	0.03	0.06	0.015	0.04	0.05	0.08	−0.012	−0.038	0.151
Proportion receiving any gender home visit	0.02	0.03	0.364	0.02	0.03	0.03	−0.016	−0.011	0.675
Proportion who attended any WASH training (s)	0.19	0.31	<0.001	0.16	0.28	0.35	−0.113	−0.184	0.144
Number of WASH training sessions attended, *n*	0.39 (0.06)	0.59 (0.05)	0.012	0.28 (0.05)	0.52 (0.06)	0.68 (0.08)	−0.245	<0.001	0.122
Proportion receiving any WASH home visit	0.07	0.13	0.037	0.06	0.12	0.13	−0.065	−0.069	0.892
Households, *n*	827	815		404	419	396	−0.086	−0.076	0.01

*Note*: This table compares household participation in different SELEVER activities (poultry, nutrition and gender and WASH‐related training sessions and follow‐up home visits) between the different treatment arms at endline. The data are presented as means with standard errors in parentheses for continuous variables or as proportions for binary variables. Trial corresponds to the first level of randomisation, while WASH substudy corresponds to the second level of randomisation. Standard errors are clustered at the commune level.

Abbreviations: WASH, Water, Sanitation and Hygiene; VVV, Village Volunteer Vaccinators.

**Table 3:** SELEVER programme uptake during lean season follow‐up, SELEVER trial and WASH‐substudy populations.

	Trial		*p* Value	WASH substudy			*p* Value Δ		
Variable	(1) Control	(2) SELEVER	(1)−(2)	(1) Control	(2) SELEVER	(3) SELEVER + WASH	(1)−(2)	(1)−(3)	(2)−(3)
Attended training on poultry, nutrition, gender and WASH	0.02	0.25	<0.001	1.19 (0.22)	4.17 (0.56)	4.53 (0.68)	<0.001	<0.001	0.983
Total number of training sessions attended, *n*	1.19 (0.22)	4.34 (0.43)	<0.001	1.19	4.17	4.53	<0.001	<0.001	0.680
Proportion who attended any poultry training (s)	0.12	0.44	<0.001	0.12	0.42	0.47	<0.001	<0.001	0.464
Number of poultry training sessions attended, *n*	0.18 (0.04)	1.5 (0.16)	<0.001	0.18 (0.04)	1.51 (0.21)	1.5 (0.24)	<0.001	<0.001	0.971
Proportion who received VVV services	0.56	0.64	0.249	0.56	0.62	0.67	0.453	0.207	0.598
Proportion who attended any nutrition training (s)	0.17	0.33	<0.001	0.17	0.34	0.33	0.002	0.004	0.871
Proportion who attended any gender training (s)	0.06	0.25	<0.001	0.06	0.25	0.25	0.001	0.001	0.961
Number of nutrition/gender training sessions attended, *n*	0.48 (0.11)	1.61 (0.21)	<0.001	0.48 (0.11)	1.54 (0.27)	1.69 (0.33)	0.001	0.001	0.718
Proportion receiving any nutrition home visit	0.04	0.11	0.003	0.04	0.09	0.13	0.043	0.006	0.302
Proportion receiving any gender home visit	0.03	0.1	0.002	0.03	0.08	0.12	0.058	0.003	0.242
Proportion who attended any WASH training (s)	0.24	0.49	<0.001	0.24	0.43	0.56	0.004	<0.001	0.056
Number of WASH training sessions attended, *n*	0.53 (0.12)	1.23 (0.13)	<0.001	0.53 (0.12)	1.13 (0.19)	1.35 (0.17)	0.011	0.001	0.385
Proportion receiving any WASH home visit	0.08	0.21	0.001	0.08	0.16	0.25	0.056	0.001	0.135
Households, *n*	346	664		346	340	324			

*Note*: This table compares household participation in different SELEVER activities (poultry, nutrition and gender and WASH‐related training practices and home visits) between the different treatment arms at endline. The data are presented as means with standard errors in parentheses for continuous variables or as proportions for binary variables. Trial corresponds to the first level of randomisation, while WASH substudy corresponds to the second level of randomisation. Standard errors are clustered at the commune level.

Abbreviations: WASH, water, sanitation and hygiene; VVV, village volunteer vaccinators.

### 3.3 Caregiver WASH‐related knowledge and practices

Caregiver knowledge of WASH and WASH–livestock‐related risks at endline was generally higher in the SELEVER group than in the control group for both mothers and fathers, with the differences across groups found to be marginally statistically significant for overall mother's knowledge and statistically significant for knowledge related to livestock risks (Table 4, Supporting Information: A2 and A3). In the WASH substudy population at endline, mothers and fathers in both SELEVER groups had higher overall hygiene knowledge scores than mothers and fathers in the control group. There was also evidence of higher knowledge scores for mothers in the SELEVER + WASH group as compared to mothers in the SELEVER group.

**Table 4:** Maternal and paternal hygiene‐related knowledge at endline, SELEVER trial.

		Endline				
	*n*	Mean (SD)	Main effect (SE)	*p* Value	Adjusted main effect (SE)	*p* Value
Mother's hygiene knowledge						
Trial						
Control	841	4.45 (2.57)				
SELEVER	831	4.77 (2.56)	0.07 (0.06)	0.249	0.10 (0.06)	0.076
WASH substudy						
Control	417	4.12 (2.51)				
SELEVER	425	4.62 (2.45)	0.11 (0.07)	0.095	0.11 (0.07)	0.130
SELEVER + WASH	406	4.93 (2.66)	0.18 (0.08)	0.027	0.22 (0.07)	0.003
Test of equality				0.239		0.013
Father's hygiene knowledge						
Trial						
Control	841	4.52 (2.88)				
SELEVER	831	4.69 (2.83)	0.04 (0.06)	0.510	0.06 (0.05)	0.486
WASH substudy						
Control	417	4.05 (3.02)				
SELEVER	425	4.74 (2.8)	0.16 (0.08)	0.055	0.14 (0.08)	0.099
SELEVER + WASH	406	4.63 (2.86)	0.14 (0.07)	0.061	0.12 (0.07)	0.089
Test of equality				0.755		0.803

*Note*: This table presents intention‐to‐treat (ITT) unadjusted and adjusted effects for hygiene knowledge for the SELEVER trial (1st level randomisation) and WASH substudy (2nd level randomisation), estimated using Poisson regression models. Variables used in the trial‐level adjusted analysis include primary education of the household head, a binary indicator for whether the household is polygamous and a binary variable indicating no chicken faeces was observed in the household compound and survey month. Variables used in the substudy‐level adjusted analysis include primary education of the household head, polygamous, water score, sanitation score, hygiene score and survey month. Household variables included in the adjusted analysis are measured at baseline. Regressions include survey weights to account for baseline sampling probabilities. Standard errors are clustered at the commune level. Test of equality refers to a post‐estimation test of the equality between the coefficients in the SELEVER and SELEVER + WASH treatment arms. Intra‐class correlation coefficients (ICC) are the mother's hygiene knowledge 0.140 and the father's hygiene knowledge 0.073.

No differences across SELEVER and control groups were apparent with regard to overall household WASH and WASH–livestock‐related practices (Table 5). Some differences were found for specific practices involving separation between livestock and children, where households in the SELEVER group were more likely to keep children separated from poultry (∆ = 0.09, 95% CI (0.03−0.15), *p* = 0.009) than households in the control group (Supporting Information: Table A4). There was also some evidence from the spot‐checks of both mothers and children appearing cleaner in the SELEVER group compared to those in the control group (Supporting Information: Table A4). In the WASH substudy population, generally, no differences across SELEVER groups were apparent (Supporting Information: Table A5).

**Table 5:** Effects of SELEVER and SELEVER + WASH interventions on WASH‐related practices, SELEVER Trial and WASH‐substudy populations.

		Endline 2020						Lean season 2019				
	*n*	Mean (SD)	Main effect (SE)	*p* Value	Adjusted main effect (SE)	*p* Value	*n*	Mean (SD)	Main effect (SE)	*p* Value	Adjusted main effect (SE)	*p* Value
WASH Score (0−16)												
Trial												
Control	842	13.68 (3.46)										
SELEVER	829	13.52 (3.43)	−0.01 (0.02)	0.565	0.01 (0.02)	0.756						
WASH substudy												
Control	417	13.65 (3.53)					343	12.93 (3.40)				
SELEVER	424	13.75 (3.48)	−0.01 (0.03)	0.784	0.00 (0.03)	0.871	342	13.99 (3.02)	0.07 (0.03)	0.031	0.07 (0.04)	0.096
SELEVER + WASH	405	13.26 (3.37)	−0.02 (0.03)	0.443	0.01 (0.02)	0.656	325	12.93 (3.40)	0.01 (0.04)	0.749	0.00 (0.04)	0.988
Test of equality				0.645		0.824				0.073		0.091
Water score (0−3)												
Trial												
Control	842	1.68 (0.72)										
SELEVER	829	1.74 (0.65)	0.03 (0.03)	0.245	0.04 (0.03)	0.224						
WASH substudy												
Control	417	1.76 (0.72)					343	1.33 (0.69)				
SELEVER	424	1.77 (0.60)	0.04 (0.03)	0.160	0.04 (0.03)	0.243	342	1.33 (0.71)	−0.01 (0.07)	0.863	−0.03 (0.10)	0.781
SELEVER + WASH	405	1.71 (0.70)	0.02 (0.03)	0.490	0.04 (0.04)	0.300	325	1.33 (0.72)	0.01 (0.08)	0.948	0.00 (0.09)	0.988
Test of equality				0.527		0.961				0.819		0.799
Sanitation score (0−3)												
Trial												
Control	842	1.31 (1.21)										
SELEVER	829	1.19 (1.22)	−0.06 (0.07)	0.427	−0.08 (0.08)	0.341						
WASH substudy												
Control	417	1.34 (1.26)					343	1.17 (1.16)				
SELEVER	424	1.28 (1.24)	−0.07 (0.09)	0.413	−0.11 (0.10)	0.294	342	1.26 (1.20)	0.02 (0.13)	0.860	−0.07 (0.13)	0.590
SELEVER + WASH	405	1.10 (1.18)	−0.04 (0.10)	0.695	−0.04 (0.10)	0.684	325	1.03 (1.18)	−0.02 (0.12)	0.846	−0.17 (0.16)	0.295
Test of equality				0.747		0.541				0.703		0.530
Hygiene score (0–10)												
Trial												
Control	842	4.90 (1.95)										
SELEVER	829	4.73 (1.96)	−0.03 (0.04)	0.391	0.01 (0.03)	0.680						
WASH substudy												
Control	417	4.77 (1.86)					343	4.35 (2.04)				
SELEVER	424	4.83 (1.92)	−0.02 (0.05)	0.742	0.01 (0.04)	0.736	342	5.26 (1.73)	0.19 (0.07)	0.008	0.13 (0.07)	0.057
SELEVER + WASH	405	4.61 (2.00)	−0.05 (0.05)	0.251	0.01 (0.03)	0.680	325	4.41 (1.98)	0.02 (0.09)	0.797	−0.04 (0.09)	0.670
Test of equality				0.493		0.981				0.016		0.021
Separation of children and animals (0−9)												
Trial												
Control	842	5.79 (1.82)										
SELEVER	829	5.85 (1.93)	0.01 (0.02)	0.793	0.02 (0.02)	0.378						
WASH substudy												
Control	417	5.79 (1.87)					343	6.07 (1.67)				
SELEVER	424	5.87 (1.96)	0.01 (0.02)	0.751	0.02 (0.02)	0.413	342	6.14 (1.55)	0.01 (0.03)	0.715	0.05 (0.03)	0.132
SELEVER + WASH	405	5.84 (1.90)	0.00 (0.02)	0.877	0.02 (0.03)	0.439	325	6.16 (1.67)	0.01 (0.03)	0.618	0.06 (0.03)	0.084
Test of equality				0.870		0.956				0.901		0.798

*Note*: This table presents intention‐to‐treat (ITT) unadjusted and adjusted effects for WASH outcomes for the SELEVER trial (1st level randomisation) and WASH substudy (2nd level randomisation), estimated using analysis of covariance (ANCOVA). Variables used in the adjusted analyses include baseline primary education of the household head, a binary indicator for whether the household is polygamous and post‐lockdown survey month at endline. Household variables included in the adjusted analysis are measured at baseline. Regressions include survey weights to account for baseline sampling probabilities. Standard errors are clustered at the commune level. Endline refers to the fourth survey round, and lean‐season to the third survey round, respectively. Test of equality refers to a postestimation test of the equality between the coefficients in the SELEVER and SELEVER + WASH treatment arms. Intra‐class correlation coefficients (ICC) are WASH score 0.156, water score 0.045, sanitation score 0.130, hygiene score 0.010 and separation of children and animals 0.021.

In the WASH substudy population in the lean‐season follow‐up, households in the SELEVER group were found to have higher overall WASH practices scores than those in control or SELEVER + WASH groups (Table 5). These differences were of small magnitude but statistically significant and appeared to be driven by results in the hygiene domain, including the observations of cleaner compounds, such as less frequent observation of soiled children's underwear and chicken faeces in the cooking area of the compound (Supporting Information: Table A6). Households in both SELEVER groups were found to be more likely to have separate areas for children and small livestock.

### 3.4 Index child morbidity symptoms and anthropometric indicators

At endline, there were no statistically significant differences across treatment groups for child morbidity or anthropometry (using adjusted means results), with the exception of a small negative effect on the height of marginal statistical significance (∆ = −0.54 cm, 95% CI (−1.09 to 0.01), *p* = 0.06) in the SELEVER + WASH group compared to the control group (Table 6). In the lean season, there was evidence of increased likelihood of diarrhoea symptoms (Δ = 0.06, 95% CI (0.02−0.10), *p* = 0.017) in the SELEVER + WASH group compared to the control group and an increase in the thinness of borderline statistical significance (Δ = 0.05, 95% CI (0.01−0.09), *p* = 0.06) in the SELEVER group compared to the control group.

**Table 6:** Effects of SELEVER and SELEVER + WASH interventions on index child morbidity symptoms and anthropometry indicators, SELEVER Trial and WASH‐substudy populations.

		Endline 2020						Lean season 2019				
	*n*	Mean (SD)	Main effect (SE)	*p* Value	Adjusted main effect (SE)	*p* Value	*n*	Mean (SD)	Main effect (SE)	*p* Value	Adjusted main effect	*p* Value
Morbidity (0−6)												
Trial												
Control	724	0.17 (0.46)										
SELEVER	709	0.16 (0.49)	−0.06 (0.22)	0.789	0.12 (0.19)	0.533						
WASH substudy												
Control	362	0.13 (0.42)					286	0.33 (0.68)				
SELEVER	370	0.19 (0.55)	0.13 (0.26)	0.624	0.23 (0.25)	0.356	297	0.35 (0.74)	0.04 (0.21)	0.836	0.05 (0.29)	0.862
SELEVER + WASH	339	0.13 (0.42)	−0.31 (0.25)	0.203	−0.12 (0.20)	0.560	272	0.34 (0.72)	0.01 (0.21)	0.966	−0.33 (0.28)	0.236
Test of equality				0.118		0.179				0.887		0.252
Diarrhoea												
Trial												
Control	724	0.01 (0.10)										
SELEVER	709	0.01 (0.11)	0.00 (0.01)	0.873	0.00 (0.01)	0.608						
WASH substudy												
Control	362	0.01 (0.12)					286	0.01 (0.08)				
SELEVER	370	0.01 (0.1)	0.00 (0.01)	0.978	0.00 (0.01)	0.733	297	0.02 (0.14)	0.01 (0.01)	0.276	0.01 (0.02)	0.494
SELEVER + WASH	339	0.01 (0.11)	0.00 (0.01)	0.779	0.00 (0.01)	0.660	272	0.06 (0.23)	0.05 (0.02)	0.002	0.06 (0.02)	0.017
Test of equality				0.787		0.872				0.046		0.141
Height												
Trial												
Control	730	114.42 (6.60)										
SELEVER	721	114.74 (6.72)	−0.41 (0.37)	0.266	−0.06 (0.30)	0.845						
WASH substudy												
Control	364	113.62 (6.53)					304	109.16 (6.58)				
SELEVER	376	115.11 (6.85)	0.18 (0.41)	0.664	0.34 (0.31)	0.285	304	110.13 (7.06)	0.21 (0.30)	0.488	0.18 (0.34)	0.604
SELEVER + WASH	345	114.33 (6.56)	−1.05 (0.40)	0.011	−0.54 (0.28)	0.061	276	109.80 (6.75)	0.00 (0.30)	0.991	0.15 (0.40)	0.716
Test of equality				0.008		0.005				0.541		0.949
Weight												
Trial												
Control	729	18.96 (2.51)										
SELEVER	719	18.99 (2.72)	−0.04 (0.14)	0.771	0.11 (0.11)	0.304						
WASH substudy												
Control	364	18.69 (2.47)					304	17.40 (2.33)				
SELEVER	374	19.15 (2.8)	0.14 (0.16)	0.407	0.20 (0.13)	0.125	304	17.64 (2.56)	−0.15 (0.09)	0.102	−0.20 (0.18)	0.278
SELEVER + WASH	345	18.8 (2.63)	−0.23 (0.16)	0.148	0.00 (0.12)	0.999	275	17.42 (2.51)	−0.24 (0.11)	0.038	−0.16 (0.21)	0.449
Test of equality				0.060		0.162				0.407		0.823
HAZ												
Trial												
Control	730	−0.79 (0.98)										
SELEVER	721	−0.75 (0.93)	−0.03 (0.06)	0.586	−0.01 (0.06)	0.914						
WASH substudy												
Control	364	−0.82 (0.95)					304	−0.95 (0.94)				
SELEVER	376	−0.72 (0.92)	0.06 (0.07)	0.436	0.06 (0.07)	0.354	304	−0.88 (0.94)	0.05 (0.06)	0.402	0.04 (0.06)	0.546
SELEVER + WASH	345	−0.78 (0.95)	−0.13 (0.06)	0.042	−0.09 (0.06)	0.111	276	−0.90 (1.00)	0.00 (0.06)	0.937	0.04 (0.08)	0.649
Test of equality				0.013		0.019				0.434		0.986
BMIZ												
Trial												
Control	729	−0.74 (0.79)										
SELEVER	719	−0.81 (0.84)	0.03 (0.05)	0.572	0.04 (0.05)	0.37						
WASH substudy												
Control	364	−0.74 (0.84)					304	−0.60 (0.79)				
SELEVER	374	−0.78 (0.82)	0.02 (0.06)	0.716	0.03 (0.06)	0.591	304	−0.65 (0.86)	−0.09 (0.06)	0.140	−0.10 (0.10)	0.317
SELEVER + WASH	345	−0.84 (0.85)	0.04 (0.06)	0.575	0.06 (0.06)	0.333	275	−0.73 (0.86)	−0.12 (0.05)	0.021	−0.09 (0.11)	0.377
Test of equality				0.832		0.695				0.606		0.959
Stunted (HAZ < −2)												
Trial												
Control	730	0.09 (0.28)										
SELEVER	721	0.09 (0.28)	0.01 (0.02)	0.618	0.00 (0.02)	0.949						
WASH substudy												
Control	364	0.09 (0.28)					304	0.14 (0.35)				
SELEVER	376	0.08 (0.27)	−0.01 (0.02)	0.530	−0.02 (0.02)	0.388	304	0.10 (0.31)	−0.02 (0.03)	0.548	−0.01 (0.04)	0.811
SELEVER + WASH	345	0.1 (0.3)	0.03 (0.02)	0.123	0.02 (0.02)	0.227	276	0.12 (0.32)	−0.01 (0.03)	0.698	−0.01 (0.04)	0.821
Test of equality				0.049		0.063				0.854		0.983
Thin (BMIZ < −2)												
Trial												
Control	729	0.06 (0.23)										
SELEVER	719	0.08 (0.27)	0.02 (0.02)	0.401	0.01 (0.02)	0.645						
WASH substudy												
Control	364	0.06 (0.24)					304	0.05 (0.21)				
SELEVER	374	0.07 (0.25)	0.00 (0.02)	0.971	0.00 (0.02)	0.902	304	0.06 (0.24)	0.03 (0.02)	0.096	0.05 (0.02)	0.060
SELEVER + WASH	345	0.09 (0.29)	0.03 (0.03)	0.239	0.03 (0.03)	0.349	275	0.07 (0.26)	0.02 (0.02)	0.276	0.04 (0.03)	0.169
Test of equality				0.314		0.362				0.876		0.722

*Note*: This table presents intention‐to‐treat (ITT) effects for child anthropometic and morbidity outcomes for the SELEVER trial (1st level randomisation) and WASH substudy (2nd level randomisation), estimated using analysis of covariance (ANCOVA). Included control variables are child age and gender. Variables used in the trial‐level adjusted analysis include the primary education of the household head, a binary indicator for whether the household is polygamous and a binary variable indicating no chicken faeces was observed in the household compound. Variables used in the substudy‐level adjusted analysis include primary education of the household head, polygamous, water score, sanitation score and hygiene score. Household variables included in the adjusted analyses are measured at baseline. Regressions include survey weights to account for baseline sampling probabilities. Standard errors are clustered at the commune level. Endline refers to the fourth survey round, and lean‐season to the third survey round, respectively. Test of equality refers to a post‐estimation test of the equality between the coefficients in the SELEVER and SELEVER + WASH treatment arms. Intra‐class correlation coefficients (ICC) are morbidity 0.013, diarrhoea 0.005, height 0.013, weight 0.018, HAZ 0.043, BMIZ 0.029, stunted 0.028 and thin 0.015.

#### 3.4.1 Younger siblings' child anthropometric indicators

In the exploratory analyses in the smaller cohort of younger siblings, at endline (Table A7), a negative effect of the SELEVER + WASH intervention was found on height (Δ = −0.8 cm, 95% CI (−1.44 to −0.16), *p* = 0.014), and HAZ (Δ = −0.19, 95% CI (−0.35 to −0.03), *p* = 0.028) compared to the control group. A negative effect of the SELEVER intervention compared to the control was also found on BMIZ (Δ = −0.18, 95% CI (−0.34 to −0.02), *p* = 0.027).

## 4 DISCUSSION

This study examined the effectiveness of a 3 year information only, nutrition‐ and gender‐sensitive poultry value chain intervention, with and without additional livestock WASH behaviour change, to reduce child morbidity symptoms and improve anthropometric indicators in children of 2−4 years of age at baseline in rural Burkina Faso. In addition, through a trial substudy, we also examined whether the treatment effects of the intervention varied by season. At endline, after 3 years of programme implementation, there was evidence that the SELEVER intervention had increased caregiver knowledge of WASH‐related livestock risks for young children. This resulted in some improvements in WASH‐related livestock practices, particularly those involving the separation of small livestock and children in the household compound. Improvements in practices were particularly apparent in the WASH substudy population during the lean season, with larger effects in the SELEVER + WASH compared to the SELEVER study arm. In the main trial, there was no evidence of positive effects of the SELEVER intervention on index children's prevalence of caregiver‐reported morbidity symptoms or of measured child anthropometric outcomes. Results from the WASH‐substudy suggest a negative, albeit very small, effect on index children's height (−0.5 cm; 95% CI (−1.09 to 0.01), marginal statistical significance *p* = 0.06) in the SELEVER + WASH group relative to both the control and SELEVER groups. Similarly, in the lean season, children in the SELEVER + WASH group had a greater likelihood of caregiver‐reported diarrhoea in the 7 days prior to the survey compared to the control group; and there was an increase in the prevalence of thinness in the SELEVER group relative to the control group (95% CI (0.01−0.09), marginal statistical significance: *p* = 0.06). Examining these results chronologically suggests that children in the SELEVER group, and particularly those in the SELEVER + WASH group, may have faced a more acute disease burden during the lean season compared to children in the control group, resulting in a small accumulated height deficit at endline. The findings from the exploratory analyses in the smaller cohort of younger siblings, a secondary reference age group in the study, were similarly suggestive of the potential negative effects of the SELEVER intervention on child anthropometry indicators, including height, HAZ and BMIZ.

There are several potential explanations for the overall modest positive to null impacts of the studies on child morbidity and anthropometry and for the suggestive negative effects of the programme on index children's height and on younger siblings' anthropometry. For interpretation, we draw on the results from the intermediary outcomes reported in this analysis and those of the analyses on the primary outcomes of the SELEVER trial reported elsewhere. First, the lack of impacts on child outcomes may be due to low programme participation. Overall programme exposure was low, peaking at 3 extra training sessions attended over the prior 12 months compared to the control group, equivalent to 1 extra training session every 3 months, which is well below the expectations of monthly training sessions. Second, our results show that although the SELEVER intervention had some positive impacts on livestock‐hygiene‐related knowledge and some livestock‐hygiene‐related practices, these gains were not sufficient to improve the overall WASH environment and reduce child morbidity. This may, in part, be due to the low participation of households in SELEVER and WASH interventions. In addition, we documented previously that the SELEVER intervention had small effects on household poultry production and no effects on household profits due to an increase in the costs of production. Thus, households had no economic returns from poultry production that they could use to invest in improving the nutrition and health of their children (Leight, Awonon, Pedehombga, Ganaba, Gelli, 2022). SELEVER also had no impacts on the micronutrient intake of index women and children (Becquey et al., 2022), another pathway through which the programme could have improved child anthropometric outcomes or reduced morbidity. Third, in terms of improvements in linear growth, index children in our sample are likely to have been less responsive to a nutrition‐sensitive programme like SELEVER compared to children in the first 1000 days of a child's life that are the critical window of opportunity for stimulating linear growth (Victora et al., 2021). This was one of the reasons why child anthropometry was a secondary outcome indicator in our study.

Another aspect that could explain the reduced differences in WASH environment between control and intervention groups at endline compared to baseline is the fact that WASH levels improved over time in both SELEVER and control groups and that increases were slightly greater in the control group compared to the to SELEVER group (Annex Table 4). This finding is in line with the scaling‐up of CLTS‐related activities as part of the national sanitation strategy adopted in Burkina Faso during the study period. Anecdotal evidence from the SELEVER process evaluation indicated that the nationwide scale‐up of CLTS may have affected the trial through the crowding‐out of resources from the national programme (in the form of subsidies towards the purchasing of slabs for latrine construction) in the SELEVER intervention areas as a result of the NGO intervention activities.

With regard to the differential effects of the SELEVER and SELEVER + WASH intervention, the descriptive analysis of baseline levels of the WASH indicators suggests that the overall WASH environment, as well as conditions in each of the WASH sub‐domains, were considerably lower in the SELEVER + WASH group compared to both SELEVER and control groups. As such, our findings may reflect the fact that improving WASH‐related behaviours and practices from a very low base may require a much larger step‐wise investment, as highlighted by the recent literature on WASH and child nutrition outcomes (Cumming et al., 2019). Much of this interpretation is speculative and further research on the health and infection biomarkers in the index children is currently underway to clarify potential mechanisms.

There are some clear programming implications of these findings. First, the study population includes households living in deep poverty and food insecurity and with very low baseline WASH conditions. In these contexts, the objectives of a nutrition‐sensitive livestock and nutrition behaviour change intervention should primarily focus on improving knowledge and practices and secondarily on child nutrition, with particular caution on avoiding unintended effects. From an implementer's perspective, a particular emphasis on increasing coverage and implementation intensity is important. However, achieving improvements in practices is also not a given without additional support, particularly in a context where financial constraints are binding and improving practices requires financial investment. To some degree, this was the case with corralling poultry in Burkina Faso, where poultry corralling was associated with increased costs of production related to the coop itself but also with regard to feed necessary to compensate for lack of scavenging opportunities for free‐roaming poultry (Leight, Awonon, Pedehombga, Ganaba, Gelli, 2022; Passarelli et al., 2021). Providing some form of transfer to encourage households to invest in improved poultry housing could be considered by programme implementers as a way to enhance potential effectiveness and avoid unintended effects (Alderman et al., 2022; Gelli et al., 2018; Olney et al., 2016). Another important takeaway from this study involves the design and implementation of the integrated intervention. On the WASH‐specific element of the SELEVER package, there was clear evidence of some cross‐over of WASH‐related activities in the SELEVER arm. Initially, the formative research undertaken as part of the study design highlighted the need for WASH messaging to feature as part of a comprehensive livestock–nutrition package, and this concept was to be tested as a separate arm in the trial implemented by a separate, independent NGO from those involved in the implementation of the other SELEVER components (Gelli, Headey, et al., 2017). However, SELEVER programme implementers responded to the formative research by also incorporating WASH messaging in the core SELEVER curriculum. As such, the SELEVER versus SELEVER + WASH comparison should arguably be interpreted as a high‐intensity versus lower‐intensity comparison. Lastly, evidence on programme exposure presented in this study and in the SELEVER process evaluation suggests that though the SELEVER programme included activities across poultry production and nutrition, these activities were implemented independently and with little coordination, following a colocation implementation model (Gelli et al., 2019). Future programme implementation should consider closer integration and coordination of implementation at the different levels involved and plan for and encourage higher implementation intensity.

### 4.1 Limitations

The main strengths of this study are the randomised design, the nested substudy with seasonal pre‐intervention baselines and the use of PIPs to understand the plausibility of programme effects. Several important limitations are also apparent. First, the measurement of intermediate outcomes on the PIPs, including WASH‐related knowledge and practices and child morbidity symptoms, involves subjective assessments that suffer from well‐documented measurement errors and a lack of validity testing. One reason for the higher prevalences of diarrhoea reported in the SELEVER groups could be that mothers in these groups are more aware of the definition of diarrhoea and thus report it more. However, the results from the anthropometry indicators, as well as the results of the SELEVER trial published elsewhere, are largely consistent with the null results that we report. The analysis of infection biomarkers that is currently underway as part of the SELEVER trial will provide more objective evidence on the complex biological processes linking animal husbandry, livestock‐related pathogen exposure and infection in young children. Another important limitation arises from the potential effects of the COVID‐19‐related lockdown that took place midway during the endline survey. However, the results from lean season analysis that pre‐dates the COVID‐related crises are largely consistent with the overall trial results. An additional limitation arises from the low level of intervention uptake in the study population, highlighting that these results reflect the low implementation fidelity rather than the potential effectiveness of livestock–WASH–gender interventions.

## 5 CONCLUSION

Integrating a livestock behaviour change WASH intervention alongside a household poultry value chain and nutrition intervention can increase the knowledge of livestock‐related hygiene risks and improve livestock‐hygiene‐related practices in rural settings in LMICs. In these contexts, and especially when programme participation is low, these types of behavioural interventions may not be sufficient to improve the health and nutritional status of young children.

## AUTHOR CONTRIBUTIONS

A. Gelli was the principal investigator on the trial. Rasmane Ganaba and Abdoulaye Pedehombga managed field operations and supervised the field data supervisors. Marco Santacroce, Loty Diop and Josue Awonon programmed the survey questionnaires and contributed to data management. Alain Hien, and Laeticia C. Toe trained the survey enumerators. Anissa Collishaw contributed to the data analysis and reporting. Elodie Becquey, Derek Headey, Hans Verhoef, Francis Ngure, Harold Alderman and Marie T. Ruel contributed to the design and interpretation. All authors contributed to, reviewed and approved this article.

## CONFLICT OF INTEREST STATEMENT

The authors declare no conflict of interest.

## Supporting information

Supporting information.

## Data Availability

The data that support the findings of this study are available from the corresponding author upon reasonable request.

## Footnotes

1 The three livestock categories were used for simplicity and were calculated using data from species‐specific variables.
