# Supplementary material for: Effects of an integrated poultry value chain, nutrition, gender and WASH intervention (SELEVER) on hygiene and child morbidity and anthropometry in Burkina Faso: A secondary outcome analysis of a cluster randomised trial
Source: Matern Child Nutr. 2023 May 27;19(4):e13528. doi: 10.1111/mcn.13528 (PMC10483954; doi:10.1111/mcn.13528)
Supplement: Supplementary file 1 — Supporting information. [file MCN-19-e13528-s001.docx]

**Effects of an integrated poultry value chain, nutrition and WASH intervention (SELEVER) on child anthropometry outcomes in Burkina Faso: A secondary outcome analysis of a cluster randomized trial.**

**Online supplementary materials, 6/9/2022**

**Research in context**

*Evidence before this study*

Before this trial, a seminal review included in the 2013 Lancet Series on Maternal and Child Nutrition highlighted that nutrition-sensitive agriculture (NSA) programs had enormous potential to accelerate progress in addressing child malnutrition. However, there was a lack of evidence on the effectiveness of these interventions due limitations in the design of interventions and evaluations. An updated review published in 2019 found that NSA programs consistently increased diet diversity and consumption of nutritious foods. From a nutrition and dietary intake perspective, interventions in livestock were of specific interest due to the high-quality protein and nutrient density of many animal-sourced food. Poultry-based programs were particularly relevant as poultry production in low income countries provides households with nutrient-rich meat and egg products, as well as cash income. However, traditional production systems present potential health and nutrition risks because poultry scavenging around household compounds may increase children's exposure to livestock-related pathogens. Exposures to faecal bacteria, for example, is associated with environmental enteric dysfunction (EED), a sub-clinical inflammation of the small intestinal lining responsible for low-grade chronic immune stimulation associated with child growth faltering. Overnight corralling of livestock within the same room as young children is also associated with elevated markers of EED and stunting. However, to date, little attention has been paid in livestock interventions to the prevention of livestock-related health risks through the promotion of improved poultry-husbandry and hygiene practices. Similarly, Water, Sanitation and Hygiene (WASH) interventions have mostly focused on reducing exposure to human faeces, even though animal faecal matter is widespread in rural, low-income contexts. Evidence gaps are especially salient when considering the large seasonal variations in diets, health and nutrition, as well as on indicators related to WASH and poultry production, documented in the context of rural Burkina Faso and the broader Sahel region. During the lean, rainy season, households often leave their compounds and relocate with their young children and livestock to live closer to their fields to protect and tend to the harvest. These temporary dwellings involve a different, more precarious WASH environment from that provided throughout the rest of the year, potentially modifying the context in which interventions operate. To our knowledge, there is also little or no evidence on how the effectiveness of integrated livestock and nutrition interventions varies by season.

*Added value of this study*

The SELEVER cluster randomised trial assessed the impact of a nutrition- and gender-sensitive poultry value chain intervention, with and without additional livestock WASH behaviour change, on child morbidity and anthropometric indices of nutrition in Burkina Faso. The intervention involved three components focussing on poultry revenue generation, diets and nutrition, and women’s empowerment domains. Intervention activities included training and behaviour change communication (BCC), with no financial or in-kind transfers provided to participants. The content of the BCC included the promotion of improved diets and basic hygiene practices. The gender component, implemented alongside the nutrition activities, included sensitization on women’s empowerment, including community level training of trainers, follow-up home visits, peer-group support and advocacy conducted by high-profile community members such as religious or traditional community leaders, and women leaders. The poultry component involved trainings on poultry husbandry, including improved housing, vaccinations, feed, financing and marketing practices. The additional WASH intervention aimed to enhance the impact of the SELEVER intervention on children’s health and nutrition status by improving the WASH environment at community and household level, reducing the risk of exposure to livestock faeces for young children. A poultry-livestock lens was applied to the Community-Led Total Sanitation (CLTS) behaviour change approach, involving three stages 1) pre-triggering (engagement with community members); 2) triggering (conducting group meeting activities to elicit emotional responses, including shame and disgust, and generate motivation to eliminate open defecation); and 3) follow-up (monitoring progress and feedback toward eliminating open defecation). In this study we show that despite low-levels of participation, these interventions increased knowledge of livestock-related risks and improved livestock-hygiene related practices. However, we found no effects on child morbidity or child anthropometric outcomes.

*Implications of all the available evidence*

Our trial provides further high-level evidence that information-only NSA interventions alone are insufficient to improve the nutritional status of young children. These findings are largely consistent with most of the evidence from the other NSA trials conducted until June 2022. Providing some form of financial transfer to encourage households to invest in improved practices could be considered to enhance the effectiveness of these types of interventions. Future NSA program implementation may also benefit from closer integration and coordination of implementation of the different program activities.

**Disruption to endline survey due to COVID-19**

The endline survey began on the 7th of March 2020 as planned, just prior to the COVID-19 pandemic. However, data collection was paused by the principal investigator on the 24th of March after consultation with the in-country research partner and the SELEVER trial steering group chair, days before the national lockdown was mandated. In mid-April the Government began easing the lockdown measures, including lifting the quarantine, reopening of roads, markets, schools and mosques. Data from WHO from that period also suggested that the number of cases remained consistently low in the period subsequent to the easing of restrictions (WHO, 2023). Adjustments were made to minimize risks of participating households and the survey team, including procuring personal protective equipment (PPE) and developing a formal standard operating procedure (SOP) on improved hygiene that was used to train enumerators during a two-day training prior to the survey restart. The SOP included measures related to PPE use, handwashing, sanitization, social-distancing and daily symptoms monitoring for both enumerators and respondents. Participating households also received guidance on hygiene practices and a set of three re-usable face masks per household, alongside the economic incentive for participating in the survey. A formal request to restart the survey was made on May 26th to the IFPRI ethics review board and senior management, as well as local IRB and health authorities in country and authorization was received on the 9th of June. Data collection resumed after a refresher enumerator training and was completed in the first week of August.

Table A1: Description of constructed scores used as outcome variables

| \| **Variable** \| **Range** \| **Description** \| \| --- \| --- \| --- \| \| Hygiene knowledge (mother, father) \| 0-18 \| Summation of responses measuring knowledge of risks associated with cohabiting with animals (0-4) and how to avoid risks posed by close contact with animals (0-4), knowledge of open defecation risks (0-4) and overcoming open defecation risks, and knowledge of why shared handwashing receptacles not recommended \| \| WATER score \| 0-3 \| Summation of binary variables indicating primary source of drinking water improved/protected, household treats water, no animal access to drinking water source \| \| SANITATION score \| 0-3 \| Summation of binary variables indicating household owns a working latrine, latrine is built in concrete, and a slab is present and in good condition \| \| HYGIENE score \| 0-10 \| Summation of binary variables indicating household has a handwashing facility, household has soap, compound appears clean, no open garbage in the concession, animals are contained, no open garbage, toilets are clean, no human feces visible, no soiled diapers in the compound, no chicken feces in the compound, no chicken feces in the kitchen area. \| \| WASH score \| 0-16 \| Summation of WATER, SANITATION and HYGIENE scores \| \| Separation of animals and children score \| 0-9 \| Summation of three sub-scores for small livestock, livestock and poultry indicating that the child does not have access to where animals spend the majority of their time, animals not kept inside the house, and distance between the animal and child at night >10m \| \| Mother's cleanliness score \| 0-4 \| Mother's hands, hair, clothing and face appear clean \| \| Child's cleanliness score \| 0-4 \| Child's hands, hair, clothing and face appear clean \| \| Morbidity score \| 0-6 \| Summation of child's caregiver-reported morbidity symptoms including vomiting, fever, cough, respiratory difficulties, diarrhea, and other illness. \| |  |  |
| --- | --- | --- | --- | --- | --- | --- | --- | --- | --- | --- | --- | --- | --- | --- | --- | --- | --- | --- | --- | --- | --- | --- | --- | --- | --- | --- | --- | --- | --- | --- | --- | --- |

Table A2: Adjusted intervention effects on maternal and paternal hygiene related knowledge at endline, SELEVER Trial.

| **Variables** | Control | Treatment | ITT effect | SE | P-value |
| --- | --- | --- | --- | --- | --- |
| Mother's hygiene knowledge |  |  |  |  |  |
| Overall hygiene knowledge (0-18) | 4.45 | 4.77 | 0.10 | 0.06 | 0.076 |
| Animal risks (0-4) | 0.88 | 0.97 | 0.16 | 0.08 | 0.051 |
| Overcoming animal risks (0-4) | 0.84 | 0.96 | 0.19 | 0.07 | 0.012 |
| Open defecation risks (0-4) | 1.01 | 1.04 | 0.06 | 0.07 | 0.378 |
| Overcoming open defecation risks (0-3) | 0.87 | 0.89 | 0.03 | 0.05 | 0.533 |
| Handwashing (0-3) | 0.85 | 0.91 | 0.08 | 0.07 | 0.243 |
| Father's hygiene knowledge |  |  |  |  |  |
| Overall hygiene knowledge (0-18) | 4.52 | 4.69 | 0.06 | 0.05 | 0.486 |
| Animal risks (0-4) | 0.90 | 1.01 | 0.15 | 0.07 | 0.116 |
| Overcoming animal risks (0-4) | 0.88 | 0.91 | 0.07 | 0.06 | 0.588 |
| Open defecation risks (0-4) | 1.03 | 1.05 | 0.03 | 0.05 | 0.768 |
| Overcoming open defecation risks (0-3) | 0.86 | 0.84 | 0.00 | 0.05 | 0.669 |
| Handwashing (0-3) | 0.84 | 0.88 | 0.07 | 0.07 | 0.522 |
| Households, n | 842 | 831 |  |  |  |

Notes: This table presents summary statistics and treatment effect estimates at endline for hygiene knowledge outcomes for the SELEVER trial (1^st^ level randomization). ITT estimates are estimated using Poisson regression models, adjusted for baseline measures of imbalanced household variables (primary education of household head, polygamous, no chicken feces in concession) and survey month. Models include survey weights to account for baseline sampling probabilities. Standard errors are clustered at the commune level. Scores are simple tallies of correct responses to different categories of knowledge questions, with higher values representing higher knowledge.

Table A3: Adjusted intervention effects on maternal and paternal hygiene related knowledge at endline, SELEVER WASH-substudy population.

|  |  |  |  | **SELEVER** | | | **SELEVER+WASH** | | | **SL vs SLW** | |
| --- | --- | --- | --- | --- | --- | --- | --- | --- | --- | --- | --- |
| **Variables** | **Control** | **SELEVER** | **SELEVER +WASH** | **ITT Effect** | **SE** | **p-value** | **ITT Effect** | **SE** | **p-value** | **Chi2** | **p-value** |
| Mother's hygiene knowledge |  |  |  |  |  |  |  |  |  |  |  |
| Overall hygiene knowledge (0-18) | 4.12 | 4.62 | 4.93 | 0.11 | 0.07 | 0.130 | 0.22 | 0.07 | 0.003 | 6.23 | 0.013 |
| Animal risks (0-4) | 0.74 | 0.95 | 1.01 | 0.21 | 0.12 | 0.077 | 0.30 | 0.12 | 0.010 | 1.70 | 0.192 |
| Overcoming animal risks (0-4) | 0.75 | 0.91 | 1.01 | 0.19 | 0.09 | 0.037 | 0.34 | 0.09 | 0.000 | 6.24 | 0.013 |
| Open defecation risks (0-4) | 0.95 | 0.99 | 1.09 | 0.04 | 0.08 | 0.621 | 0.19 | 0.08 | 0.014 | 6.01 | 0.014 |
| Overcoming open defecation risks (0-3) | 0.84 | 0.85 | 0.93 | -0.01 | 0.05 | 0.921 | 0.15 | 0.06 | 0.009 | 10.83 | 0.001 |
| Handwashing (0-3) | 0.83 | 0.92 | 0.89 | 0.10 | 0.10 | 0.325 | 0.12 | 0.10 | 0.218 | 0.06 | 0.810 |
| Father's hygiene knowledge |  |  |  |  |  |  |  |  |  |  |  |
| Overall hygiene knowledge (0-18) | 4.05 | 4.74 | 4.63 | 0.14 | 0.08 | 0.099 | 0.12 | 0.07 | 0.089 | 0.06 | 0.803 |
| Animal risks (0-4) | 0.78 | 0.99 | 1.03 | 0.22 | 0.12 | 0.057 | 0.25 | 0.10 | 0.017 | 0.09 | 0.759 |
| Overcoming animal risks (0-4) | 0.77 | 0.91 | 0.91 | 0.13 | 0.11 | 0.233 | 0.15 | 0.09 | 0.120 | 0.03 | 0.858 |
| Open defecation risks (0-4) | 0.95 | 1.04 | 1.05 | 0.08 | 0.07 | 0.232 | 0.10 | 0.07 | 0.195 | 0.03 | 0.866 |
| Overcoming open defecation risks (0-3) | 0.80 | 0.86 | 0.82 | 0.07 | 0.08 | 0.356 | 0.02 | 0.06 | 0.707 | 0.66 | 0.415 |
| Handwashing (0-3) | 0.75 | 0.93 | 0.82 | 0.18 | 0.10 | 0.058 | 0.09 | 0.08 | 0.291 | 0.92 | 0.337 |
| Households, n | 417 | 425 | 406 |  |  |  |  |  |  |  |  |

Notes: This table presents summary statistics and treatment effect estimates at endline for hygiene knowledge outcomes for the WASH sub-study (2^nd^ level randomization). Abbreviations: ITT, intent to treat; SE, standard error. ITT estimates are estimated using Poisson regression models, adjusted for baseline measures of imbalanced household variables (primary education of household head, polygamous, water score, sanitation score, hygiene score) and survey month. Models include survey weights to account for baseline sampling probabilities. Standard errors are clustered at the commune level. Scores are simple tallies of correct responses to different categories of knowledge questions, with higher values representing higher knowledge.

Table A4: Adjusted intervention effects on WASH practices at endline, SELEVER Trial.

|  | **Control** | | **Treatment** | |  |  |  |
| --- | --- | --- | --- | --- | --- | --- | --- |
| **Variable** | **Baseline** | **Endline** | **Baseline** | **Endline** | **ITT effect** | **SE** | **P value** |
| WASH Score (0-16) | 13.23 | 13.69 | 13.27 | 13.52 | 0.01 | 0.02 | 0.756 |
| Water(0-3) | 1.16 | 1.68 | 1.18 | 1.74 | 0.04 | 0.03 | 0.224 |
| Non-surface | 0.63 | 0.71 | 0.70 | 0.80 | 0.08 | 0.05 | 0.084 |
| Treated | 0.12 | 0.19 | 0.09 | 0.18 | -0.04 | 0.04 | 0.311 |
| No animal access | 0.42 | 0.79 | 0.40 | 0.76 | 0.01 | 0.04 | 0.760 |
| Sanitation(0-3) | 0.96 | 1.31 | 0.86 | 1.19 | -0.08 | 0.08 | 0.341 |
| Latrine | 0.45 | 0.59 | 0.40 | 0.53 | -0.03 | 0.05 | 0.528 |
| Cement | 0.20 | 0.24 | 0.19 | 0.21 | -0.03 | 0.03 | 0.288 |
| Slab | 0.31 | 0.48 | 0.28 | 0.45 | -0.03 | 0.05 | 0.528 |
| Hygiene(0-10) | 4.45 | 4.90 | 4.40 | 4.73 | 0.01 | 0.03 | 0.659 |
| Handwashing facility | 0.04 | 0.05 | 0.03 | 0.03 | -0.02 | 0.01 | 0.090 |
| Have soap | 0.02 | 0.02 | 0.00 | 0.01 | -0.01 | 0.01 | 0.172 |
| Clean appearance | 0.40 | 0.48 | 0.42 | 0.47 | 0.05 | 0.04 | 0.130 |
| No garbage | 0.39 | 0.48 | 0.42 | 0.46 | 0.05 | 0.04 | 0.250 |
| No free roaming animals | 0.73 | 0.64 | 0.72 | 0.65 | -0.01 | 0.03 | 0.719 |
| Clean toilet | 0.26 | 0.33 | 0.27 | 0.27 | -0.05 | 0.04 | 0.189 |
| No human feces visible | 0.88 | 0.93 | 0.87 | 0.91 | -0.01 | 0.02 | 0.726 |
| No soiled underwear | 0.80 | 0.91 | 0.79 | 0.92 | 0.02 | 0.02 | 0.338 |
| No chicken feces in concession | 0.32 | 0.37 | 0.26 | 0.36 | 0.05 | 0.04 | 0.204 |
| No chicken feces in kitchen | 0.62 | 0.69 | 0.64 | 0.65 | -0.01 | 0.03 | 0.863 |
| Sep. of children & animals (0-9) | 6.66 | 5.79 | 6.83 | 5.85 | 0.02 | 0.02 | 0.378 |
| Child-small livestock | 2.14 | 1.82 | 2.26 | 1.91 | 0.03 | 0.03 | 0.310 |
| Child-livestock | 2.52 | 2.39 | 2.49 | 2.27 | -0.03 | 0.03 | 0.261 |
| Child-Poultry | 2.00 | 1.59 | 2.08 | 1.67 | 0.09 | 0.03 | 0.009 |
| Mother's appearance clean (0-4) | 2.87 | 3.01 | 2.69 | 3.10 | 0.08 | 0.04 | 0.072 |
| Mother's hands | 0.75 | 0.81 | 0.69 | 0.83 | 0.06 | 0.04 | 0.099 |
| Mother's hair | 0.63 | 0.58 | 0.59 | 0.63 | 0.08 | 0.04 | 0.083 |
| Mother's clothing | 0.71 | 0.76 | 0.66 | 0.78 | 0.06 | 0.04 | 0.119 |
| Mother's face | 0.79 | 0.87 | 0.75 | 0.86 | 0.03 | 0.03 | 0.423 |
| Child's appearance clean | 1.65 | 1.42 | 1.40 | 1.53 | 0.07 | 0.08 | 0.351 |
| Child's hands | 0.33 | 0.33 | 0.25 | 0.32 | 0.00 | 0.03 | 0.931 |
| Child's hair | 0.48 | 0.39 | 0.44 | 0.43 | 0.05 | 0.04 | 0.191 |
| Child's clothing | 0.36 | 0.30 | 0.30 | 0.32 | 0.02 | 0.03 | 0.528 |
| Child's face | 0.48 | 0.41 | 0.42 | 0.46 | 0.05 | 0.04 | 0.179 |
| Households, n | 842 | 842 | 829 | 829 |  |  |  |

Notes: This table presents summary statistics and adjusted intention-to-treat (ITT) effects for WASH outcomes for the SELEVER trial (1^st^ level randomization). ITT effects are estimated using analysis of covariance (ANCOVA). Specifically, ITT effects for count variables are estimated using Poisson regression models, while ITT effects for binary variables are estimated using linear probability models. All models control for the outcome of interest and imbalanced household variables (primary education of household head and polygamous) measured at baseline and include survey weights to account for baseline sampling probabilities. Month of endline survey is also included to account for disruptions to data collection as a result of a national lockdown in March 2020 due to Covid-19. Standard errors are clustered at the commune level. Variables and scores are coded such that higher values indicate preferred outcomes. Baseline and endline refer to the first and fourth survey rounds, respectively.

Table A5: Adjusted intervention effects on WASH practices at endline, SELEVER WASH-substudy population.

|  | **Control** | | **SELEVER** | | **SELEVER+WASH** | | **SELEVER** | | | **SELEVER+WASH** | | | **SL vs SL+ WASH** | |
| --- | --- | --- | --- | --- | --- | --- | --- | --- | --- | --- | --- | --- | --- | --- |
| **Variable** | **Baseline** | **Endline** | **Baseline** | **Endline** | **Baseline** | **Endline** | **ITT effect** | **SE** | **P-Value** | **ITT effect** | **SE** | **P-Value** | **F/Chi2** | **P-Value** |
| **WASH Score (0-16)** | **13.16** | **13.65** | **13.76** | **13.75** | **12.75** | **13.26** | **0.00** | **0.03** | **0.871** | **0.01** | **0.02** | **0.656** | **0.05** | **0.824** |
| Water (0-3) | 1.24 | 1.76 | 1.29 | 1.77 | 1.07 | 1.71 | 0.04 | 0.03 | 0.243 | 0.04 | 0.04 | 0.300 | 0.00 | 0.961 |
| Non-surface | 0.72 | 0.79 | 0.74 | 0.83 | 0.65 | 0.78 | 0.10 | 0.04 | 0.021 | 0.06 | 0.06 | 0.330 | 0.44 | 0.511 |
| Treated | 0.11 | 0.18 | 0.11 | 0.15 | 0.07 | 0.22 | -0.09 | 0.04 | 0.025 | 0.01 | 0.06 | 0.806 | 3.53 | 0.065 |
| No animal access | 0.42 | 0.79 | 0.44 | 0.80 | 0.35 | 0.71 | 0.04 | 0.04 | 0.411 | -0.02 | 0.05 | 0.736 | 0.98 | 0.326 |
| **Sanitation (0-3)** | **0.93** | **1.34** | **1.08** | **1.28** | **0.62** | **1.10** | **-0.11** | **0.10** | **0.294** | **-0.04** | **0.10** | **0.684** | **0.37** | **0.541** |
| Latrine | 0.42 | 0.57 | 0.47 | 0.55 | 0.32 | 0.50 | -0.04 | 0.06 | 0.465 | -0.02 | 0.06 | 0.781 | 0.21 | 0.652 |
| Cement | 0.20 | 0.27 | 0.25 | 0.24 | 0.12 | 0.18 | -0.04 | 0.04 | 0.364 | -0.03 | 0.04 | 0.395 | 0.01 | 0.923 |
| Slab | 0.31 | 0.50 | 0.37 | 0.48 | 0.18 | 0.42 | -0.04 | 0.06 | 0.500 | -0.02 | 0.05 | 0.703 | 0.12 | 0.727 |
| **Hygiene (0-10)** | **4.46** | **4.77** | **4.52** | **4.83** | **4.27** | **4.61** | **0.01** | **0.04** | **0.736** | **0.01** | **0.03** | **0.680** | **0.00** | **0.981** |
| Handwashing facility | 0.03 | 0.05 | 0.03 | 0.04 | 0.02 | 0.03 | -0.02 | 0.01 | 0.171 | -0.02 | 0.02 | 0.183 | 0.01 | 0.942 |
| Have soap | 0.01 | 0.03 | 0.01 | 0.01 | 0.00 | 0.01 | -0.01 | 0.01 | 0.274 | -0.01 | 0.01 | 0.166 | 0.08 | 0.773 |
| Clean appearance | 0.38 | 0.43 | 0.48 | 0.50 | 0.35 | 0.43 | 0.06 | 0.05 | 0.210 | 0.05 | 0.04 | 0.195 | 0.05 | 0.818 |
| No garbage | 0.38 | 0.43 | 0.43 | 0.47 | 0.40 | 0.45 | 0.04 | 0.05 | 0.461 | 0.06 | 0.04 | 0.192 | 0.11 | 0.746 |
| No free roaming animals | 0.78 | 0.67 | 0.70 | 0.61 | 0.73 | 0.70 | -0.02 | 0.04 | 0.536 | 0.00 | 0.04 | 0.962 | 0.37 | 0.544 |
| Clean toilet | 0.25 | 0.29 | 0.33 | 0.29 | 0.22 | 0.26 | -0.06 | 0.05 | 0.197 | -0.04 | 0.04 | 0.360 | 0.20 | 0.657 |
| No human feces visible | 0.86 | 0.95 | 0.86 | 0.94 | 0.87 | 0.88 | 0.01 | 0.02 | 0.551 | -0.03 | 0.02 | 0.184 | 3.17 | 0.080 |
| No soiled underwear | 0.80 | 0.92 | 0.78 | 0.93 | 0.80 | 0.91 | 0.02 | 0.02 | 0.261 | 0.02 | 0.03 | 0.569 | 0.10 | 0.753 |
| No chicken feces in concession | 0.32 | 0.33 | 0.27 | 0.37 | 0.24 | 0.34 | 0.04 | 0.05 | 0.388 | 0.06 | 0.04 | 0.193 | 0.10 | 0.752 |
| No chicken feces in kitchen | 0.65 | 0.68 | 0.64 | 0.68 | 0.63 | 0.61 | 0.00 | 0.03 | 0.943 | -0.01 | 0.04 | 0.812 | 0.03 | 0.865 |
| **Sep of children & animals (0-9)** | **6.53** | **5.79** | **6.87** | **5.87** | **6.80** | **5.84** | **0.02** | **0.02** | **0.413** | **0.02** | **0.03** | **0.439** | **0.00** | **0.956** |
| Child-small livestock (0-3) | 2.10 | 1.84 | 2.27 | 1.90 | 2.25 | 1.93 | 0.03 | 0.04 | 0.433 | 0.04 | 0.04 | 0.301 | 0.17 | 0.683 |
| Child-livestock (0-3) | 2.46 | 2.32 | 2.50 | 2.29 | 2.49 | 2.24 | -0.03 | 0.03 | 0.374 | -0.03 | 0.03 | 0.216 | 0.05 | 0.819 |
| Child-poultry (0-3) | 1.97 | 1.63 | 2.11 | 1.68 | 2.06 | 1.67 | 0.09 | 0.03 | 0.005 | 0.08 | 0.04 | 0.064 | 0.04 | 0.838 |
| **Mother's appearance clean (0-4)** | **2.92** | **2.89** | **2.69** | **3.12** | **2.69** | **3.09** | **0.09** | **0.05** | **0.089** | **0.06** | **0.05** | **0.183** | **0.24** | **0.622** |
| Mother's hands | 0.75 | 0.75 | 0.70 | 0.83 | 0.68 | 0.81 | 0.08 | 0.04 | 0.065 | 0.03 | 0.04 | 0.407 | 0.98 | 0.327 |
| Mother's hair | 0.64 | 0.62 | 0.59 | 0.61 | 0.59 | 0.66 | 0.06 | 0.05 | 0.277 | 0.09 | 0.04 | 0.029 | 0.49 | 0.487 |
| Mother's clothing | 0.71 | 0.71 | 0.66 | 0.81 | 0.66 | 0.75 | 0.09 | 0.05 | 0.064 | 0.03 | 0.04 | 0.456 | 1.94 | 0.169 |
| Mother's face | 0.82 | 0.81 | 0.74 | 0.86 | 0.76 | 0.87 | 0.03 | 0.04 | 0.435 | 0.02 | 0.04 | 0.578 | 0.03 | 0.863 |
| **Child's appearance clean (0-4)** | **1.73** | **1.50** | **1.54** | **1.67** | **1.25** | **1.38** | **0.12** | **0.09** | **0.191** | **0.01** | **0.08** | **0.860** | **1.91** | **0.167** |
| Child's hands | 0.33 | 0.31 | 0.30 | 0.37 | 0.19 | 0.27 | 0.03 | 0.04 | 0.402 | -0.04 | 0.04 | 0.219 | 4.42 | 0.040 |
| Child's hair | 0.50 | 0.42 | 0.45 | 0.46 | 0.42 | 0.41 | 0.06 | 0.04 | 0.213 | 0.04 | 0.03 | 0.293 | 0.27 | 0.605 |
| Child's clothing | 0.40 | 0.34 | 0.34 | 0.35 | 0.26 | 0.28 | 0.04 | 0.04 | 0.327 | 0.00 | 0.03 | 0.930 | 1.63 | 0.207 |
| Child's face | 0.50 | 0.44 | 0.46 | 0.49 | 0.38 | 0.42 | 0.07 | 0.04 | 0.145 | 0.03 | 0.04 | 0.442 | 0.58 | 0.449 |
| Households, n | 417 | 417 | 424 | 424 | 405 | 405 |  |  |  |  |  |  |  |  |

Notes: This table presents summary statistics and adjusted intention-to-treat (ITT) effects for WASH outcomes for the WASH sub-study (2^nd^ level randomization). As above, ITT effects are estimated using analysis of covariance (ANCOVA), with treatment effects estimated using Poisson regression models for count variables and using linear probability models for binary variables. All models control for the outcome of interest and imbalanced household variables (primary education of household head and polygamous) measured at baseline and include survey weights to account for baseline sampling probabilities. Month of endline survey is also included to account for disruptions to data collection as a result of a national lockdown in March 2020 due to Covid-19. Standard errors are clustered at the commune level. Variables and scores are coded such that higher values indicate preferred outcomes. Baseline and endline refer to the first and fourth survey rounds, respectively.

Table A6: Adjusted intervention effects on WASH practices at lean-season follow-up, SELEVER WASH sub-study population.

|  | **Control** | | **SELEVER** | | **SELEVER+WASH** | | **SELEVER** | | | **SELEVER+WASH** | | | **SL vs SL+ WASH** | |
| --- | --- | --- | --- | --- | --- | --- | --- | --- | --- | --- | --- | --- | --- | --- |
| **Variable** | **Baseline** | **Endline** | **Baseline** | **Endline** | **Baseline** | **Endline** | **ITT effect** | **SE** | **P-Value** | **ITT effect** | **SE** | **P-Value** | **F/Chi2** | **P-Value** |
| **WASH Score (0-16)** | **13.15** | **12.93** | **13.55** | **13.99** | **12.61** | **12.93** | **0.07** | **0.04** | **0.096** | **0.00** | **0.04** | **0.988** | **2.86** | **0.091** |
| **Water(0-3)** | **1.31** | **1.33** | **1.42** | **1.33** | **1.17** | **1.33** | **-0.03** | **0.10** | **0.781** | **0.00** | **0.09** | **0.988** | **0.07** | **0.799** |
| Non-surface | 0.72 | 0.74 | 0.74 | 0.82 | 0.69 | 0.75 | 0.04 | 0.07 | 0.558 | 0.07 | 0.07 | 0.301 | 0.16 | 0.692 |
| Treated | 0.09 | 0.09 | 0.10 | 0.08 | 0.09 | 0.12 | 0.02 | 0.03 | 0.552 | 0.06 | 0.04 | 0.200 | 0.77 | 0.386 |
| No animal access | 0.50 | 0.50 | 0.57 | 0.42 | 0.39 | 0.46 | -0.10 | 0.10 | 0.304 | -0.13 | 0.12 | 0.294 | 0.06 | 0.804 |
| **Sanitation(0-3)** | **0.96** | **1.18** | **1.08** | **1.26** | **0.76** | **1.03** | **-0.07** | **0.13** | **0.590** | **-0.17** | **0.16** | **0.295** | **0.39** | **0.530** |
| Latrine | 0.43 | 0.55 | 0.50 | 0.56 | 0.38 | 0.46 | -0.09 | 0.07 | 0.198 | -0.15 | 0.07 | 0.034 | 0.66 | 0.420 |
| Cement | 0.19 | 0.19 | 0.22 | 0.22 | 0.15 | 0.17 | 0.02 | 0.05 | 0.693 | -0.04 | 0.05 | 0.438 | 1.03 | 0.315 |
| Slab | 0.35 | 0.43 | 0.37 | 0.48 | 0.24 | 0.40 | 0.02 | 0.08 | 0.782 | -0.02 | 0.09 | 0.828 | 0.26 | 0.611 |
| **Hygiene(0-10)** | **4.20** | **4.35** | **4.27** | **5.26** | **3.93** | **4.41** | **0.13** | **0.07** | **0.057** | **-0.04** | **0.09** | **0.670** | **5.21** | **0.022** |
| Handwashing facility | 0.03 | 0.02 | 0.01 | 0.02 | 0.02 | 0.01 | 0.00 | 0.02 | 0.938 | -0.02 | 0.02 | 0.123 | 2.72 | 0.106 |
| Have soap | 0.01 | 0.00 | 0.01 | 0.01 | 0.00 | 0.00 | -0.01 | 0.01 | 0.328 | -0.01 | 0.01 | 0.329 | 0.32 | 0.575 |
| Clean appearance | 0.32 | 0.38 | 0.36 | 0.53 | 0.31 | 0.39 | 0.08 | 0.07 | 0.253 | -0.02 | 0.07 | 0.783 | 2.36 | 0.131 |
| No garbage | 0.35 | 0.33 | 0.33 | 0.49 | 0.28 | 0.36 | 0.14 | 0.08 | 0.073 | 0.00 | 0.08 | 0.962 | 2.93 | 0.094 |
| No free roaming animals | 0.77 | 0.66 | 0.74 | 0.73 | 0.74 | 0.67 | 0.06 | 0.07 | 0.367 | -0.03 | 0.07 | 0.655 | 2.27 | 0.139 |
| Clean toilet | 0.27 | 0.30 | 0.27 | 0.36 | 0.23 | 0.27 | 0.04 | 0.06 | 0.469 | -0.04 | 0.07 | 0.598 | 1.78 | 0.189 |
| No human feces visible | 0.86 | 0.88 | 0.88 | 0.96 | 0.89 | 0.89 | 0.03 | 0.03 | 0.336 | -0.02 | 0.05 | 0.642 | 1.47 | 0.232 |
| No soiled underwear | 0.82 | 0.87 | 0.80 | 0.95 | 0.79 | 0.85 | 0.08 | 0.04 | 0.040 | -0.06 | 0.05 | 0.260 | 12.74 | 0.001 |
| No chicken feces in concession | 0.23 | 0.25 | 0.29 | 0.43 | 0.22 | 0.31 | 0.07 | 0.08 | 0.426 | -0.01 | 0.08 | 0.928 | 0.74 | 0.393 |
| No chicken feces in kitchen | 0.54 | 0.65 | 0.59 | 0.80 | 0.45 | 0.65 | 0.12 | 0.07 | 0.072 | 0.04 | 0.09 | 0.689 | 1.26 | 0.268 |
| **Sep of children & animals (0-9)** | **6.67** | **6.07** | **6.78** | **6.14** | **6.75** | **6.16** | **0.05** | **0.03** | **0.132** | **0.06** | **0.03** | **0.084** | **0.07** | **0.798** |
| Child-small livestock | 2.18 | 1.93 | 2.21 | 2.03 | 2.24 | 2.02 | 0.11 | 0.05 | 0.018 | 0.10 | 0.05 | 0.043 | 0.09 | 0.768 |
| Child-livestock | 2.43 | 2.41 | 2.53 | 2.46 | 2.48 | 2.43 | 0.06 | 0.04 | 0.134 | 0.06 | 0.04 | 0.113 | 0.02 | 0.893 |
| Child-poultry | 2.06 | 1.73 | 2.03 | 1.66 | 2.04 | 1.72 | -0.03 | 0.06 | 0.646 | 0.01 | 0.06 | 0.847 | 0.50 | 0.481 |
| Households, n | 343 | 343 | 342 | 342 | 325 | 235 |  |  |  |  |  |  |  |  |

Notes: This table presents summary statistics and adjusted intention-to-treat (ITT) effects for WASH outcomes for the WASH sub-study (2^nd^ level randomization) in the lean season. As above, ITT effects are estimated using analysis of covariance (ANCOVA), with treatment effects estimated using Poisson regression models for count variables and using linear probability models for binary variables. All models control for the outcome of interest and imbalanced household variables (primary education of household head and polygamous) measured at baseline and include survey weights to account for baseline sampling probabilities. Standard errors are clustered at the commune level. Variables and scores are coded such that higher values indicate preferred outcomes. Baseline and endline refer to the second and third survey rounds, the lean season baseline and follow-up.

Table A7 Effects of SELEVER and SELEVER+WASH interventions on younger sibling’s anthropometry indicators, SELEVER Trial and WASH-sub study populations.

|  |  |  | **Endline** | | | | |  | **Lean season** | | | | |
| --- | --- | --- | --- | --- | --- | --- | --- | --- | --- | --- | --- | --- | --- |
|  |  | **n** | **Mean (SD)** | **Main effect** | **p value** | **Adjusted main effect (SE)** | **p value** | **n** | **Mean (SD)** | **Main effect (SE)** | **p value** | **Adjusted main effect** | **p value** |
| **Height** | | |  |  |  |  |  |  |  |  |  |  |  |
| Trial | Control | 142 | 100.41 (5.38) |  |  |  |  |  |  |  |  |  |  |
|  | SELEVER | 302 | 99.81 (6.10) | -0.57 (0.79) | 0.474 | -0.26 (0.38) | 0.489 |  |  |  |  |  |  |
| WASH sub-study | Control | 142 | 100.41 (5.38) |  |  |  |  | 117 | 95.03 (5.33) |  |  |  |  |
|  | SELEVER | 138 | 101.76 (5.55) | 0.64 (0.67) | 0.348 | 0.28 (0.47) | 0.556 | 103 | 96.66 (5.90) | 0.74 (0.63) | 0.243 | 0.38 (0.63) | 0.556 |
|  | SELEVER+WASH | 164 | 98.20 (6.08) | -1.55 (0.93) | 0.101 | -0.8 (0.33) | 0.014 | 142 | 93.78 (4.84) | -0.59 (0.62) | 0.349 | -1.51 (0.76) | 0.052 |
|  | SEL=SEL+WASH |  |  |  | 0.017 |  | 0.037 |  |  |  | 0.060 |  | 0.013 |
|  |  |  |  |  |  |  |  |  |  |  |  |  |  |
| **Weight** | | |  |  |  |  |  |  |  |  |  |  |  |
| Trial | Control | 142 | 14.97 (1.83) |  |  |  |  |  |  |  |  |  |  |
|  | SELEVER | 303 | 14.66 (2.05) | -0.31 (0.21) | 0.148 | -0.25 (0.14) | 0.085 |  |  |  |  |  |  |
| WASH sub-study | Control | 142 | 14.97 (1.83) |  |  |  |  | 117 | 13.66 (1.72) |  |  |  |  |
|  | SELEVER | 138 | 15.06 (1.93) | -0.12 (0.19) | 0.550 | -0.21 (0.17) | 0.228 | 103 | 13.96 (2.10) | 0.08 (0.23) | 0.743 | 0.10 (0.28) | 0.738 |
|  | SELEVER+WASH | 165 | 14.35 (2.09) | -0.46 (0.26) | 0.087 | -0.33 (0.19) | 0.097 | 143 | 13.38 (1.74) | -0.27 (0.20) | 0.168 | -0.32 (0.25) | 0.207 |
|  | SEL=SEL+WASH |  |  |  | 0.178 |  | 0.586 |  |  |  | 0.149 |  | 0.194 |
|  |  |  |  |  |  |  |  |  |  |  |  |  |  |
| **HAZ** | |  |  |  |  |  |  |  |  |  |  |  |  |
| Trial | Control | 142 | -1.01 (1.00) |  |  |  |  |  |  |  |  |  |  |
|  | SELEVER | 302 | -1.24 (1.13) | -0.10 (0.13) | 0.442 | -0.08 (0.09) | 0.394 |  |  |  |  |  |  |
| WASH sub-study | Control | 142 | -1.01 (1.00) |  |  |  |  | 117 | -1.20 (1.14) |  |  |  |  |
|  | SELEVER | 138 | -0.96 (1.03) | 0.14 (0.12) | 0.258 | 0.03 (0.11) | 0.769 | 103 | -1.10 (1.23) | 0.27 (0.16) | 0.106 | 0.01 (0.17) | 0.933 |
|  | SELEVER+WASH | 164 | -1.47 (1.16) | -0.30 (0.15) | 0.052 | -0.19 (0.08) | 0.028 | 142 | -1.50 (0.92) | -0.11 (0.15) | 0.462 | -0.39 (0.17) | 0.029 |
|  | SEL=SEL+WASH |  |  |  | 0.012 |  | 0.058 |  |  |  | 0.012 |  | 0.022 |
|  |  |  |  |  |  |  |  |  |  |  |  |  |  |
| **BMIZ** | |  |  |  |  |  |  |  |  |  |  |  |  |
| Trial | Control | 142 | -0.41 (0.87) |  |  |  |  |  |  |  |  |  |  |
|  | SELEVER | 303 | -0.46 (0.87) | -0.07 (0.08) | 0.360 | -0.07 (0.07) | 0.316 |  |  |  |  |  |  |
| WASH sub-study | Control | 142 | -0.41 (0.87) |  |  |  |  | 117 | -0.25 (0.91) |  |  |  |  |
|  | SELEVER | 138 | -0.63 (0.87) | -0.23 (0.08) | 0.009 | -0.18 (0.08) | 0.027 | 103 | -0.40 (0.81) | -0.18 (0.09) | 0.043 | -0.02 (0.14) | 0.906 |
|  | SELEVER+WASH | 165 | -0.33 (0.85) | 0.05 (0.07) | 0.459 | 0.04 (0.07) | 0.619 | 143 | -0.16 (0.84) | -0.03 (0.07) | 0.722 | 0.15 (0.12) | 0.225 |
|  | SEL=SEL+WASH |  |  |  | 0.001 |  | 0.021 |  |  |  | 0.108 |  | 0.256 |
|  |  |  |  |  |  |  |  |  |  |  |  |  |  |
| **Stunted** | |  |  |  |  |  |  |  |  |  |  |  |  |
| Trial | Control | 142 | 0.16 (0.36) |  |  |  |  |  |  |  |  |  |  |
|  | SELEVER | 302 | 0.21 (0.41) | 0.03 (0.04) | 0.445 | 0.03 (0.04) | 0.398 |  |  |  |  |  |  |
| WASH sub-study | Control | 142 | 0.16 (0.36) |  |  |  |  | 117 | 0.22 (0.42) |  |  |  |  |
|  | SELEVER | 138 | 0.11 (0.31) | -0.04 (0.04) | 0.269 | -0.03 (0.04) | 0.575 | 103 | 0.18 (0.38) | -0.07 (0.05) | 0.166 | 0.03 (0.06) | 0.646 |
|  | SELEVER+WASH | 164 | 0.29 (0.45) | 0.10 (0.05) | 0.076 | 0.07 (0.04) | 0.092 | 142 | 0.33 (0.47) | 0.07 (0.05) | 0.207 | 0.13 (0.06) | 0.058 |
|  | SEL=SEL+WASH |  |  |  | 0.023 |  | 0.072 |  |  |  | 0.022 |  | 0.205 |
|  |  |  |  |  |  |  |  |  |  |  |  |  |  |
| **Thin** | |  |  |  |  |  |  |  |  |  |  |  |  |
| Trial | Control | 142 | 0.03 (0.18) |  |  |  |  |  |  |  |  |  |  |
|  | SELEVER | 303 | 0.03 (0.17) | -0.01 (0.02) | 0.798 | 0.00 (0.02) | 0.875 |  |  |  |  |  |  |
| WASH sub-study | Control | 142 | 0.03 (0.18) |  |  |  |  | 117 | 0.03 (0.17) |  |  |  |  |
|  | SELEVER | 138 | 0.03 (0.16) | -0.01 (0.02) | 0.761 | -0.01 (0.03) | 0.639 | 103 | 0.01 (0.11) | -0.02 (0.02) | 0.218 | -0.06 (0.04) | 0.172 |
|  | SELEVER+WASH | 165 | 0.03 (0.17) | 0.00 (0.02) | 0.847 | 0.00 (0.02) | 0.909 | 143 | 0.02 (0.12) | -0.03 (0.01) | 0.059 | -0.05 (0.05) | 0.304 |
|  | SEL=SEL+WASH |  |  |  | 0.876 |  | 0.614 |  |  |  | 0.498 |  | 0.849 |

Notes: This table presents intention-to-treat (ITT) effects for anthropometic outcomes for the SELEVER trial (1^st^ level randomization) and WASH sub-study (2^nd^ level randomization) for younger siblings aged 6-24 months at baseline, estimated using analysis of covariance (ANCOVA). Included control variables are child age and gender. Variables used in the trial-level adjusted analysis include primary education of the household head, a binary indicator for whether the household is polygamous, and a binary variable indicating no chicken feces was observed in the household compound. Variables used in the sub-study-level adjusted analysis include primary education of the household head, polygamous, water score, sanitation score and hygiene score. Regressions include survey weights to account for baseline sampling probabilities. Standard errors are clustered at the commune level. Endline refers to the fourth survey round, and lean season to third survey round, respectively.
